# Focal adhesion kinase signaling – tumor vulnerabilities and clinical opportunities

**DOI:** 10.1242/jcs.261723

**Published:** 2024-07-22

**Authors:** David D. Schlaepfer, Marjaana Ojalill, Dwayne G. Stupack

**Affiliations:** University of California, San Diego, Department of Obstetrics, Gynecology, and Reproductive Sciences, Moores Cancer Center, Division of Gynecologic Oncology, 3855 Health Sciences Dr., La Jolla, CA 92098, USA

**Keywords:** Focal adhesion kinase, Cell survival, Tumor recurrence, Chemotherapy resistance, Small-molecule inhibitor, Clinical trial

## Abstract

Focal adhesion kinase (FAK; encoded by *PTK2*) was discovered over 30 years ago as a cytoplasmic protein tyrosine kinase that is localized to cell adhesion sites, where it is activated by integrin receptor binding to extracellular matrix proteins. FAK is ubiquitously expressed and functions as a signaling scaffold for a variety of proteins at adhesions and in the cell cytoplasm, and with transcription factors in the nucleus. FAK expression and intrinsic activity are essential for mouse development, with molecular connections to cell motility, cell survival and gene expression. Notably, elevated FAK tyrosine phosphorylation is common in tumors, including pancreatic and ovarian cancers, where it is associated with decreased survival. Small molecule and orally available FAK inhibitors show on-target inhibition in tumor and stromal cells with effects on chemotherapy resistance, stromal fibrosis and tumor microenvironment immune function. Herein, we discuss recent insights regarding mechanisms of FAK activation and signaling, its roles as a cytoplasmic and nuclear scaffold, and the tumor-intrinsic and -extrinsic effects of FAK inhibitors. We also discuss results from ongoing and advanced clinical trials targeting FAK in low- and high-grade serous ovarian cancers, where FAK acts as a master regulator of drug resistance. Although FAK is not known to be mutationally activated, preventing FAK activity has revealed multiple tumor vulnerabilities that support expanding clinical combinatorial targeting possibilities.

## Teaser

**Summary:** Summary of 30 years of focal adhesion kinase (FAK) research with a view of the ongoing clinical testing of small-molecule FAK inhibitors.

## Introduction

Focal adhesion kinase (FAK; encoded by as *PTK2*) is a cytoplasmic protein tyrosine kinase that is recruited to and activated at sites of integrin clustering and actomyosin tension generation (McLean et al., 2005; Parsons, 2003; Schaller, 2010). Integrins do not possess intrinsic catalytic activity, yet integrin-initiated signals impact cell division, movement and survival, and promote tumor progression (Hamidi and Ivaska, 2018; Pang et al., 2023). The discovery of highly tyrosine-phosphorylated proteins at cell adhesion sites [where transmembrane integrin receptors link the extracellular matrix (ECM) to the intracellular actin cytoskeleton] (Burridge, 2017) provided initial support for the notion of integrin-associated tyrosine kinase activity. Indeed, various kinases are clustered at cell adhesion sites (Cooper and Giancotti, 2019); among these, phosphorylation events by the non-receptor protein tyrosine kinases FAK and Src relay integrin signal transmission within cells.

Loss of FAK expression or mutational inhibition of FAK activity results in embryonic lethality in mice at gastrulation (Ilic et al., 1995). FAK-null fibroblasts exhibit static cell adhesions, loss of cell polarity and slow directional cell movement in response to integrin ligands or soluble growth factor stimuli (Mitra et al., 2005; Sieg et al., 2000; Tomar and Schlaepfer, 2009). Although integrin–matrix binding is required for normal adhesion-dependent cell proliferation, this does not necessarily require FAK expression or activity (Lim et al., 2010a). Rather, FAK is associated with survival signaling and the suppression of anoikis, a type of programmed cell death after a cell detaches from ECM (Frisch et al., 1996).

In this Review, we discuss the various roles for FAK in normal and tumor cells, with an emphasis on cell phenotypes associated with the inhibition of FAK activity. We highlight recent studies regarding biomechanical and conformational-induced FAK activation, roles for FAK in the nucleus and developments in targeting kinase-independent FAK functions. Although FAK can be activated by alternative splicing (Toutant et al., 2002), gains in the FAK gene locus (*PTK2*) are also associated with FAK activation in both mouse and human tumors (Diaz Osterman et al., 2019). Elevated FAK signaling can also occur in parallel with oncogene activation, and FAK signaling is associated with adaptive tumor resistance to chemotherapy. As FAK functions as a master regulator of chemotherapy resistance, we discuss the tumor-intrinsic and -extrinsic effects of small-molecule FAK inhibitors on sensitizing cells to chemotherapies, reducing tumor fibrosis and counteracting immune evasion. Additionally, we will highlight ongoing FAK inhibitor phase II and III clinical trials in high- and low-grade serous ovarian cancers, respectively, that are incorporating combinatorial approaches to address unmet clinical needs.

## FAK protein structure and activation

### FAK protein domains and scaffolding functions

**Fig. 1.**
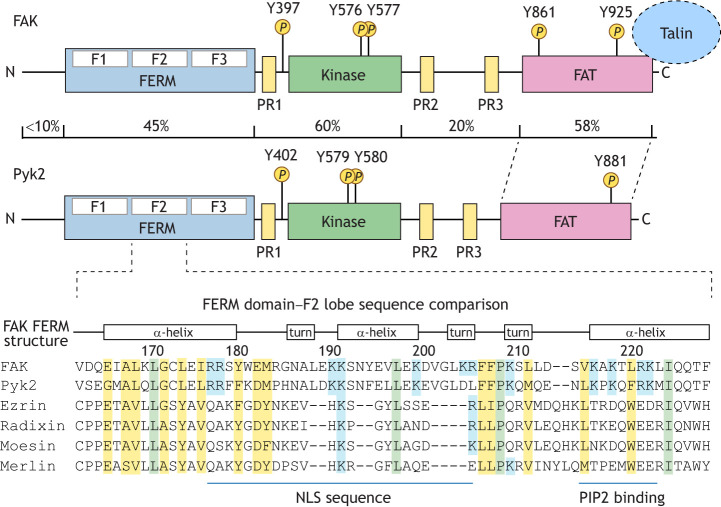
**Commonalities and differences between FAK and Pyk2.** FAK (115 kDa) shares a similar domain architecture to Pyk2 (110 kDa), with sequence identity greatest in the N-terminal band 4.1, ezrin, radixin, moesin homology (FERM), central kinase and focal adhesion targeting (FAT) domains. Conserved proline-rich (PR1, PR2 and PR3) regions serve as binding sites for Src-homology 3 (SH3) domain-containing proteins. Phosphorylation of FAK at Y397 creates a binding site for the Src SH2 domain, and subsequent Src phosphorylation FAK at Y576 and Y577 within the kinase domain activation loop leads to the formation of an active FAK–Src signaling complex. Phosphorylation sites are conserved in Pyk2 at Y402, Y579 and Y580. Src-mediated phosphorylation of FAK at Y925 and Pyk2 at Y881 creates SH2-binding sites for the Grb2 adaptor protein. FAK and Pyk2 FAT domains bind to the focal adhesion protein paxillin, with FAK also binding talin. Pyk2 contains a putative non-canonical calmodulin binding site in the C-terminal region that is not present in FAK. Alignment of the FERM F2 lobe residues is shown, with conservation between FAK and Pyk2 in residues important for nuclear localization and phosphatidylinositol 4,5-bisphosphate (PIP2) lipid binding. Conserved basic (blue), identical hydrophobic (green), and conserved residues in multiple FERM domains (yellow) are highlighted.

FAK is a 120 kDa protein comprising an N-terminal band 4.1, ezrin, radixin, moesin homology (FERM) domain, a kinase domain, an unstructured region containing proline-rich motifs and a C-terminal focal adhesion-targeting (FAT) domain (Parsons, 2003) (Fig. 1). FAK can form higher-order structures via dimerization (FERM–FERM and FAT–FAT domain interactions). Within FAK, the FERM domain also makes intramolecular regulatory contacts with the FAK kinase domain to stabilize an inactive (‘closed’) conformation (Le Coq et al., 2022). FERM and FAT domains, as well as proline-rich regions, also bind to various structural and signaling proteins that contribute to FAK scaffolding function at adhesions (Schaller, 2010).

FERM domains are present in over 50 different proteins and generally comprise three lobes termed F1, F2 and F3 (Frame et al., 2010). For FAK, activation requires the release of FERM-mediated inhibitory constraints followed by induced protein dimerization. For the FAK FERM F1 lobe, point mutations or N-terminal truncations result in elevated FAK tyrosine (Y) phosphorylation at Y397 in cells (Cohen and Guan, 2005). The FERM F1 lobe blocks access to Y397 (Lietha et al., 2007), a key regulatory site that lies within a short linker region between the FERM and kinase domains (Marlowe et al., 2019).

### FAK activation requires multiple steps

**Fig. 2.**
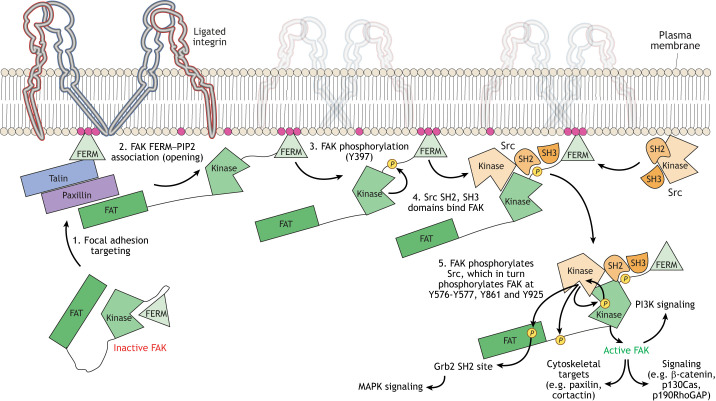
**Model illustrating FAK activation steps at adhesions.** (1) Inactive FAK is recruited to focal adhesion structures via the association of the FAK FAT domain with adaptor and adhesion-associated proteins, such as paxillin and talin. (2) The FAK FERM domain binds to PIP2 at the membrane, freeing a loop containing FAK Y397 and an SH3-binding site. (3) FAK then autophosphorylates the Y397 site, creating an SH2-binding site. (4) Src family kinases, including Src itself, bind tightly to the free loop by reinforcing SH2 and SH3 interactions. (5) FAK-scaffolded Src then phosphorylates FAK on residues Y576 and Y577 in the kinase activation loop, fully activating the kinase activity of FAK. FAK reinforces Src activity by phosphorylating Src at Y416. Src also phosphorylates FAK Y861 and Y925, creating sites to trigger signaling cascades, such as the MAPK and PI3K pathways.

As discussed above, FAK exists in a closed conformation that is inactive due to multiple steric constraints (Fig. 2), another of which is mediated by FAK FERM F2 lobe intramolecular binding to the FAK kinase domain (Lietha et al., 2007). This conformational restraint is released by the FAK FERM F2 lobe binding to phosphatidylinositol 4,5-bisphosphate [PI(4,5)P_2_], which also brings FAK to membranes (Cai et al., 2008; Goni et al., 2014) or endosomes (Alanko and Ivaska, 2016; Takahashi et al., 2021) (Figs 1 and 2). FAK can become ‘primed’ by first acquiring an ‘open’ conformation, and becomes fully active upon Y576 and Y577 kinase domain phosphorylation.

Alternatively, ezrin protein binding to the FAK FERM F1 lobe (Poullet et al., 2001) or a rise in intracellular pH (Choi et al., 2013) increases FAK Y397 phosphorylation (and FAK activity), likely by causing similar conformational changes. Cryogenic electron microscopy analyses between auto-inhibited and membrane-bound FAK have revealed conformational changes and oligomeric FAK assembly on membranes (Acebron et al., 2020), supporting the notion that FAK signaling is likely mediated by multimeric protein complexes.

### FAK and Pyk2

Whereas FAK is ubiquitously expressed in mice and in humans, the FAK-related Pyk2 protein tyrosine kinase (encoded by *PTK2B*) is co-expressed with FAK in distinct cells and tissues (Gil-Henn et al., 2024). Pyk2 possesses similar domain structures and regulatory phosphorylation sites to FAK and binds many of the same adaptor proteins (Fig. 1). However, mouse knockout studies have revealed that Pyk2 does not rescue the motility defects of FAK-null cells in part due to reduced focal adhesion localization and lack of binding to talin family proteins (hereafter talin) compared to FAK (Klingbeil et al., 2001; Lawson et al., 2012). Nevertheless, interpretations of FAK-null phenotypes can be complicated by potential effects of Pyk2 signaling (Sieg et al., 1998; Weis et al., 2008). Although elevated Pyk2 expression and tyrosine phosphorylation can enhance carcinoma, glioblastoma and chronic lymphocytic leukemia tumor growth (Gil-Henn et al., 2024; Sbrana et al., 2023), it remains unclear at a fundamental level how Pyk2-generated signals are distinct from those induced by FAK (Thomas et al., 2019).

## FAK signaling

### Co-operative and non-redundant roles of FAK and Src

FAK expression, FAK Y397 phosphorylation and intrinsic FAK activity are essential for mouse embryogenesis (Heim et al., 2018; Ilic et al., 1995; Lim et al., 2010a). During cell adhesion to matrix proteins, following FAK Y397 cis- or trans-phosphorylation, full catalytic FAK activation occurs upon Src family tyrosine kinase-mediated phosphorylation of FAK at Y576 and Y577 in the kinase activation loop (Lietha et al., 2007). Src-family kinases contain SH2 and SH3 domains, which bind to phosphorylated FAK at Y397 and to a nearby FAK proline-rich motif, respectively. This stabilizes the Src–FAK signaling complex (Fig. 2) (Marlowe et al., 2019; Schaller, 2010), with both proteins bound in open and kinase-active conformations. Notably, many functions of a FAK–Src complex are likely to be Src specific, as Src inhibition prevents the tyrosine phosphorylation of several FAK-bound proteins (Dawson et al., 2021; Mitra and Schlaepfer, 2006; Sulzmaier et al., 2014), as well as FAK Y925 phosphorylation, which acts as a signaling linkage to the mitogen-activated protein kinase cascade through Grb2 (Mitra and Schlaepfer, 2006; Mitra et al., 2006b). FAK Y861 is also phosphorylated by Src, although the function of this phosphorylation remains unclear.

**Fig. 3.**
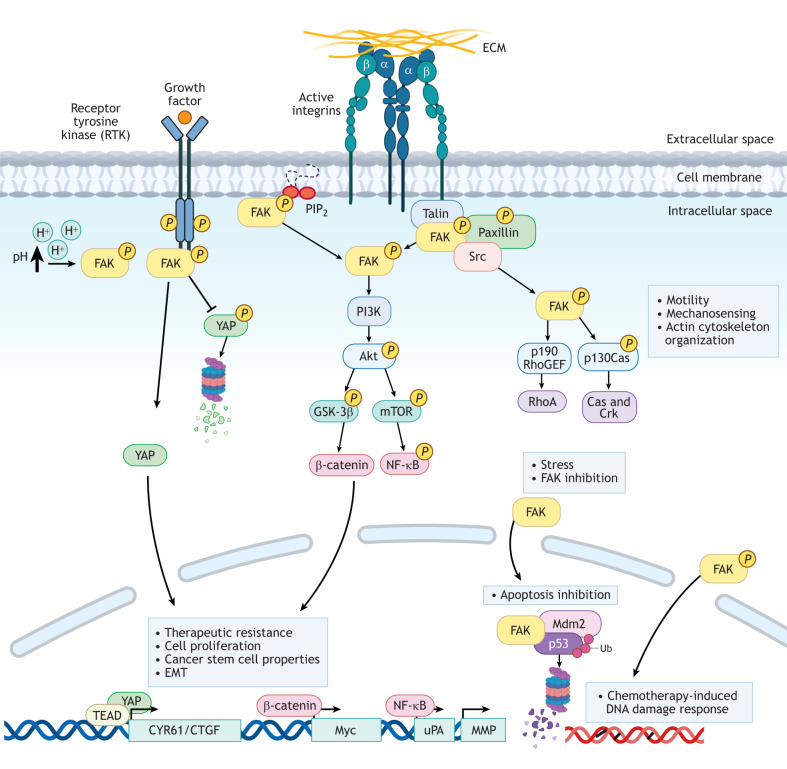
**FAK signaling as an active kinase or scaffolding protein.** FAK can be activated by several mechanisms, including (but not limited to) adhesion receptor integrins bound to ECM, receptor tyrosine kinases (RTKs), changes in intracellular pH (H^+^) and in response to cellular stress like chemotherapy exposure. Once activated, FAK as a kinase generates signals through phosphorylation cascades. FAK also functions as a scaffolding protein for complexes in the cytoplasm and nucleus, which can affect cell survival via inhibition of apoptosis and induction of proliferation. FAK participates in the regulation of cell motility, mechanosensing, cytoskeleton organization, proliferation, inhibition of apoptosis and therapeutic resistance, in addition to supporting cancer stem cells and epithelial-to-mesenchymal phenotypes. Created using BioRender.com. Ub, polyubiquitylation. p190 RhoGEF is also known as ARHGEF28.

FAK–Src signaling at integrin clusters is reinforced by tension-based FAK activation (discussed below), or alternatively, FAK and Src can be differentially activated by growth factor stimuli (Sulzmaier et al., 2014) (Fig. 3). For example, cytoplasmic FAK is rapidly recruited to endothelial cell–cell contacts upon vascular endothelial growth factor (VEGF) stimulation, which results in cell–cell junction breakdown and increased vascular permeability, which is crucial for the initiation of angiogenesis, achieved in part through vascular endothelial cadherin and β-catenin tyrosine phosphorylation (Chen et al., 2012; Jean et al., 2014). Thus, the role of Src in VEGF signaling is different from FAK (Kim et al., 2009). Additionally, as β-catenin is a central component of the Wingless and Int-1 (Wnt) signaling cascade, FAK-specific activation of β-catenin and Wnt signaling also represents a major pathway supporting neoplastic transformation (Fig. 3) (Chen et al., 2018; Diaz Osterman et al., 2019; Gao et al., 2015; Shang et al., 2019; Worthmuller and Ruegg, 2020). Finally, as revealed by FAK activity-dependent biosensors, temporal differences in FAK and Src activation are observed at adhesions (Wu et al., 2016), further supporting co-operative and overlapping, but non-redundant roles for these kinases.

### FAK as a tensional biosensor

Advances in the integrin signaling field have revealed that adhesion proteins, such as talin and vinculin, are part of a flexible force transduction unit where integrins and the actin cytoskeleton sense and transmit changes in mechanical forces (Carisey et al., 2013; Grashoff et al., 2010). FAK does not bind directly to integrins and is recruited to newly forming adhesion sites in part through the FAK C-terminal FAT domain binding to paxillin and talin (Kadare et al., 2015). Although FAK FAT binding to talin occurs independently of paxillin (Lawson et al., 2012) (Fig. 1), and is not essential for initial FAK Y397 phosphorylation at nascent adhesions, prevention of FAK and talin binding disrupts force-activated FAK signaling affecting cell migration (Zhou et al., 2021). In addition, reduced FAK–talin binding attenuates Yes-associated protein (YAP; also known as YAP1) nuclear localization and transcriptional activity, providing a signaling linkage between tension and gene transcription (Holland et al., 2024). FAK activity has been shown to increase in a manner that is proportional to the substrate rigidity (0.2 to 40 kPa) in experiments using a phosphorylation biosensor (Seong et al., 2013). Interestingly, FAK-associated mechanosensitive signaling proteins, such as p130Cas (also known as BCAR1) and downstream Rac GTPase activation also transduce matrix stiffness cues into changes in cell cycle progression (Bae et al., 2014). Together, these studies support the notion that FAK signaling can facilitate or regulate crosstalk between tensional, motility and proliferative regulatory signaling pathways.

Using atomic force microscopy and fluorescently tagged FAK proteins, one study revealed that applied tensile force to cells results in stepwise conformational changes in FAK domains (Bauer et al., 2019). Current models propose that FAK protein unfolding and clustering at nascent adhesions leads to FAK activation (Le Coq et al., 2022). However, in cells with mature adhesions, applied tension precedes full FAK activation (Li et al., 2023). These findings are consistent with earlier studies showing that FAK Y397 phosphorylation is greater when breast carcinoma cells are on stiff matrices than when on soft matrices, with stiff matrix enhancing integrin to FAK signaling and tumor progression (Levental et al., 2009). Taken together, FAK activation can precede or follow tensional changes in cells in nascent and mature adhesions, respectively. Moreover, tensional FAK activation is associated with endothelial, cardiac muscle, bone and tumor cells pathologies (as reviewed in Urciuoli and Peruzzi, 2020).

### Kinase-dependent and -independent roles for nuclear FAK

Key cell survival functions of FAK are also mediated via protein interactions with the FAK FERM domain, which contains a nuclear localization sequence in the FAK FERM F2 lobe (Lim et al., 2008) (Fig. 1). Notably, FAK FERM-mediated nuclear localization plays important roles during development and in tumor progression (Frame et al., 2010). During early mouse development, FAK loss is lethal and is associated with p53 tumor suppressor activation, with induction of p21CIP-dependent mesenchymal cell cycle arrest (Ilic et al., 1995). p21CIP1 (also known as CDKN1A) is a cyclin-dependent kinase inhibitor, and primary fibroblast proliferation in the absence of FAK is enabled by p21CIP1 knockout; in these cells, FAK FERM nuclear localization functions as a binding scaffold for p53 and the Mdm2 ubiquitin E3 ligase in the nucleus, resulting in p53 ubiquitylation and degradation (Lim et al., 2008). This nuclear FAK FERM scaffolding function does not require intrinsic FAK activity (Lim et al., 2010a) and is conserved in Pyk2 (Lim et al., 2010b). FAK-FERM binding to different ubiquitin E3 ligases can also modulate levels of other target proteins via proteasomal regulation (Canel et al., 2017, 2023; Jeong et al., 2019; Lim et al., 2012) (Fig. 3). Although FAK contains nuclear import and export consensus motifs, the temporal regulation of nuclear-, cytoplasmic- and adhesion-associated FAK in cells remains unresolved.

## FAK in tumor progression – intrinsic effects

FAK signaling (both scaffold and kinase activity) connects to a variety of different downstream intracellular pathways (Fig. 3). In this section, we highlight the broad range of cellular commonalities and phenotypes arising from FAK activation and/or FAK catalytic inhibition.

### Promoting cell invasion and metastasis

Both FAK and Pyk2 function as hubs for signaling networks that promote an invasive tumor cell phenotype. FAK is canonically known to promote cell motility at focal adhesions (Mitra et al., 2005) and Pyk2 signaling supports formation of invadopodia, protease-containing cell projections that create stromal escape conduits for tumor cells (Genna and Gil-Henn, 2018; Genna et al., 2018; Mierke et al., 2017). However, in breast carcinoma cells, FAK signaling also enhances matrix metalloproteinase (Hsia et al., 2003; Wu et al., 2005) and urokinase plasminogen activator expression (Mitra et al., 2006a), which both act in the tumor microenvironment to facilitate matrix degradation and cell invasion.

Interestingly, in melanoma cells, preventing FAK activity or inhibiting paxillin binding to FAK reduces cell migration and prevents cell-associated proteolytic activity, but paradoxically increases invadopodia structure formation (Mousson et al., 2021). This reciprocal regulation has also been observed upon FAK depletion in breast carcinoma cells, where FAK knockdown cells exhibit decreased tyrosine-phosphorylated protein abundance at focal adhesions, with increased protein tyrosine phosphorylation at invadopodia (Chan et al., 2009). As such, it is tempting to speculate that upon FAK loss, this reciprocal regulation of adhesion and invadopodia by tyrosine phosphorylation in tumor cells involves Pyk2.

### Enhancing cell survival and chemotherapy resistance

**Fig. 4.**
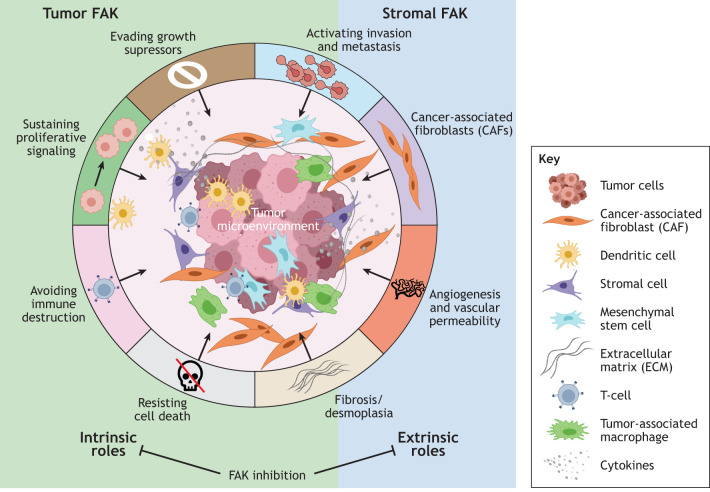
**FAK-intrinsic and -extrinsic hallmarks in tumors.** FAK participates in several of the ‘hallmarks of cancer’ (Hanahan and Weinberg, 2000). Tumor phenotypes [evading growth suppressors, activating invasion and metastasis, angiogenesis and vascular permeability, cancer-associated fibroblasts (CAFs), fibrosis and desmoplasia, avoiding immune destruction, resisting cells death and sustaining proliferative signaling] are dependent on either FAK expression or activity. Genetic deletion studies in mice have identified tumor intrinsic (left side, highlighted in green) processes that require functional FAK in tumor cells, and those regulated by FAK expression or activity in somatic tissues (right side, highlighted in blue). Small-molecule inhibitors of FAK, used *in vivo*, act upon both intrinsic and extrinsic FAK dependencies. Created using BioRender.com.

A key regulatory point of tumor metastasis is the ability of tumor cells to survive anoikis following loss of a supportive ECM environment. Facilitating such survival, membrane-targeted FAK promotes cell transformation and anchorage-independent growth, which is dependent on FAK Y397 phosphorylation and kinase activity (Frisch et al., 1996). FAK also activates other signaling pathways, such as phosphoinositide 3-kinase (PI3K) and Wnt signaling pathways, to facilitate cell survival (Zouq et al., 2009). Notably, genetic or pharmacological FAK inhibition selectively promotes tumor cell apoptosis only in anchorage-independent conditions (Tancioni et al., 2014; Tanjoni et al., 2010). Interestingly, FAK activation can also occur in a tumor-intrinsic manner upon cellular adaptive resistance to chemotherapy (Diaz Osterman et al., 2019; Kessler et al., 2019; Taylor and Schlaepfer, 2018). Although the molecular mechanism(s) of FAK activation in response to chemotherapy stress are not known, stress-induced FAK activation supports cell survival (Shapiro et al., 2014), a cancer stem cell-like phenotype (Kolev et al., 2017), and chemotherapy resistance (Diaz Osterman et al., 2019) (Fig. 4).

As well as its cytoplasmic functions, nuclear FAK is also implicated in cancer cell survival and chemotherapy resistance. In vascular smooth muscle and endothelial cells, pharmacological FAK inhibition is associated with FAK nuclear accumulation (Jeong et al., 2022, 2021; Murphy et al., 2023). However, antibodies against phosphorylated FAK Y397 stain both cytoplasmic and nucleoli structures in human breast tumors (Tancioni et al., 2015). Accordingly, wild-type but not kinase-inactive K454R FAK biochemically co-fractionates with nucleoli in breast carcinoma cells grown under anchorage-independent conditions (Tancioni et al., 2015). Together, these results support the notion that FAK nuclear localization is not necessarily associated with inhibition or activation of FAK Y397 phosphorylation. Moreover, as genetically inactive (FAK K454R) FAK can become localized to the nucleus, the processes of FAK activation or nuclear localization are likely independently regulated.

One target of nuclear FAK is the p53 tumor suppressor protein, and this linkage extends to squamous cell carcinoma (SCC) tumors (Pifer et al., 2023). In a mouse SCC model, nuclear FAK activity was found to promote chemokine transcription and subsequent alterations in the tumor-immune microenvironment (Serrels et al., 2015, 2017), contrasting with the absence of FAK in the nuclei of non-transformed keratinocytes. Although specific phosphorylation target(s) of nuclear FAK have not been identified, FAK-dependent changes in chromatin accessibility are associated with altered transcription factor binding to gene regulatory sites (Griffith et al., 2021). Taken together, the control of nuclear FAK distribution is complex and regulatory mechanisms might differ in normal versus transformed cells. Moreover, nuclear FAK signaling in tumors likely has important roles in promoting anoikis and chemotherapy resistance in a manner that does not directly involve signaling from adhesions.

## FAK in tumor progression – extrinsic effects

### Regulation of angiogenesis and vascular permeability

There is a strong link between FAK, VEGF signaling and the regulation of angiogenesis during development and tumor progression (Roy-Luzarraga and Hodivala-Dilke, 2016; Stone et al., 2014). For example, inhibition of FAK activity in breast cancer cells reduces VEGF expression and prevents angiogenesis (Mitra et al., 2006b). Additionally, as FAK inactivation is lethal during development and is associated with blood vessel morphogenesis defects (Lim et al., 2010a), conditional models of FAK inactivation have been developed. In blood vessel endothelial cells (ECs) of adult mice, conditional EC FAK loss supported normal tumor growth and angiogenesis (Weis et al., 2008), whereas in a related EC FAK knockout model, FAK loss resulted in partial inhibitory effects on tumor size and neovascularization, albeit using less-aggressive implanted tumor cells (Tavora et al., 2010).

One explanation for these results is that EC FAK and Pyk2 share overlapping functions. Pyk2 is expressed in FAK-null ECs (Weis et al., 2008), and like FAK, Pyk2 is activated by VEGF and promotes EC sprouting (Shen et al., 2011). Mechanistically, intrinsic FAK activity and FAK Y397 phosphorylation support EC angiogenesis (Pedrosa et al., 2019). FAK Y397 is homologous to Pyk2 Y402 and both serve as Src SH2-binding sites when phosphorylated. Distinguishing EC FAK from Pyk2 function *in vivo* will likely require the creation of a double FAK and Pyk2 conditional knockout mouse model.

Meanwhile, the use of an EC-specific inducible FAK kinase-dead (KD) knock-in mouse (EC-FAK-KD) has revealed a new role for FAK in facilitating VEGF-stimulated vascular permeability (Chen et al., 2012). VEGF promotes tension-independent FAK activation, FAK localization to EC cell–cell junctions and binding of the FAK FERM domain to the vascular endothelial cadherin (VE-cadherin) cytoplasmic tail. Additionally, direct FAK phosphorylation of β-catenin at Y142 facilitates VE-cadherin–β-catenin dissociation and EC junctional breakdown (Chen et al., 2012). In the EC-FAK-KD model and in breast tumor-bearing mice treated with an oral FAK inhibitor, orthotopic melanoma metastasis was attenuated without effects on tumor growth (Jean et al., 2014). As tumor cell spread through blood vessels requires cell intravasation and extravasation across EC barriers, these results are consistent with the role of EC FAK activity in controlling barrier function. Further, a recent study confirmed that FAK loss in ECs prevents pancreatic tumor metastasis in the presence of the chemotherapeutic drug gemcitabine, without affecting tumor size (Roy-Luzarraga et al., 2022). Taken together, FAK connections to VEGF signaling are both tumor extrinsic and EC intrinsic (Fig. 4).

### Stromal FAK in promoting chemotherapy resistance and fibrosis

Despite limited effects of EC FAK knockout or FAK inhibitor treatment on tumor growth, genetic loss of FAK in ECs can sensitize tumors to DNA-damaging therapies (Tavora et al., 2014). This finding was extended to a different EC-FAK-KD model using melanoma tumors (Newport et al., 2022). Additionally, low EC FAK Y397 phosphorylation levels within human breast tumors (i.e. reduced EC-associated protective signals) have been correlated with tumor chemosensitivity and increased patient survival (Roy-Luzarraga et al., 2020). Although it is hypothesized that a FAK protective signal is in part due to changes in EC cytokine production, this link remains incompletely defined.

In pancreatic ductal adenocarcinoma (PDAC), cancer-associated fibroblasts (CAFs) play key roles in tumor progression and chemoresistance, and form a physical ECM barrier limiting immune cell infiltration (Qin et al., 2024). Elevated FAK Y397 phosphorylation in CAFs is a poor prognostic marker for disease-free PDAC patient survival (Zaghdoudi et al., 2020). Expression of the FAK-KD mutant in CAFs reduced PDAC tumor fibrosis with decreased collagen matrix production (Zaghdoudi et al., 2020). Notably, development of resistance to FAK inhibition over time has been shown to be associated with the reversal of an activated CAF phenotype in a mouse PDAC model (Jiang et al., 2020). In a breast cancer model, FAK inactivation in CAFs reduced tumor cell metastasis but not tumor size (Wu et al., 2020), which was associated with alterations in CAF exosome production that impacted tumor cell migration.

In a *BRAF*-mutated melanoma mouse model, adaptive resistance alterations of CAFs leads to matrix production, remodeling and subsequent FAK activation within tumor cells to promote melanoma survival (Hirata et al., 2015). In addition, in breast cancer, chemotherapy-induced stromal collagen IV upregulation promotes tumor FAK activation and an invasive phenotype (Fatherree et al., 2022). Together, these studies point to a potential reciprocal signaling loop occurring with CAF FAK activation, increased collagen matrix production, and stromal stiffening leading to tensional activation of FAK in tumor cells. However, this type of stromal stiffness linkage to FAK may be tumor type specific.

## Omics of FAK in cancer

### FAK gene amplification

Early studies evaluating FAK levels in human tumor cell lines noted that several lung, breast and colon carcinoma cells exhibited FAK gene (*PTK2*) gains (Agochiya et al., 1999). The *PTK2* gene, in the Chr8q24.3 locus, is frequently part of a DNA amplification region in breast, uterine, cervical and ovarian female cancers (Kaveh et al., 2016). Genome-wide association studies identified the Chr8q24 region as a high-grade serous ovarian cancer (HGSOC) susceptibility locus that included *MYC* at Chr8q24.2 (Goode et al., 2010). Notably, over 70% of HGSOC tumors contain gains at both the *PTK2* and *MYC* loci (Diaz Osterman et al., 2019), and gains in Chr8q24 are associated with poor patient survival (Gorringe et al., 2010).

Additionally, *PTK2* gene copy number gains parallel increased *PTK2* mRNA and FAK protein levels in HGSOC tumors, and elevated *PTK2* mRNA levels are associated with decreased overall patient survival (Diaz Osterman et al., 2019). Proteomic analyses have revealed that FAK protein and FAK Y397 phosphorylation are elevated in HGSOC tumors relative to surrounding stromal tissue (Zhang et al., 2016). Interestingly, proliferative serous tubal intraepithelial carcinoma, a precursor of HGSOC, exhibits gains in Chr8q24 (Wang et al., 2024). Additionally, Chr8q24.3-associated circulating tumor DNA is detected in over 75% of plasma blood samples from individuals with HGSOC at the time of diagnosis (Paracchini et al., 2021). Together, these results support the notion that Chr8q24.3 gains and elevated FAK expression occur early in HGSOC tumor initiation and might be considered a potential biomarker associated with aggressive tumors.

### Spontaneous FAK gene gains in a mouse ovarian tumor model

ID8 mouse ovarian tumor cells are a highly used syngeneic but slow-growing tumor model (Roby et al., 2000). To select more aggressive cells, ID8 cells were intraperitoneally injected into C57Bl6 mice, re-collected by peritoneal wash after 40 days, and grown *ex vivo* under anchorage-independent conditions (Ward et al., 2013). Exome DNA sequencing of the recovered cells revealed that there were thousands of nucleotide changes compared to parental cells, but less than 1% of these changes were located in exons and none were predicted to be oncogenic- or tumor suppressor-associated changes (Diaz Osterman et al., 2019). Instead, comparisons revealed gains or losses across murine chromosomes, and the loci for murine *Kras*, *Myc* and *Ptk2* (FAK) genes exhibited two-to-three-fold gains compared to levels in parental ID8 cells. These *in vivo* and unbiased selected cells were renamed KMF to denote the Kras, Myc and FAK gene gains that also co-occur in HGSOC tumors (Diaz Osterman et al., 2019). Interestingly, spontaneous gains in regions of Chr15D1-4, the mouse equivalent of Chr8q24, were also detected in fallopian tube-derived tumor cells from genetically engineered mouse models (Maniati et al., 2020). Together, gains in the loci for Myc and FAK, common in murine and human ovarian tumors, are multi-factorial contributors to ovarian tumorigenesis.

Despite the above findings, genetic inactivation of FAK in KMF cells did not alter growth in adherent culture (Diaz Osterman et al., 2019). Instead, comparison of KMF FAK-null and FAK-reconstituted cells showed that FAK expression and activity promotes anchorage-independent cell survival, Wnt–β-catenin signaling and elevated intrinsic resistance to cisplatin chemotherapy (Diaz Osterman et al., 2019). Although these results point to an oncogenic role for FAK, the signals promoting FAK activation in KMF cells and in HGSOC tumors remain incompletely defined. One possibility is that FAK may be activated by alternative splicing. Alternatively spliced FAK transcripts (termed Box 6, Box 7 and Box 28, named for the number of inserted residues around the FAK Y397 site) are present in neuronal tissue transcripts and these FAK isoforms exhibit elevated FAK Y397 phosphorylation compared to wild-type FAK (Toutant et al., 2002). In pancreatic and breast neuroendocrine tumors, a high frequency of FAK mRNAs with alternative splicing are detected and these are associated with elevated FAK Y397 phosphorylation and more aggressive tumors (Xie et al., 2023). In colorectal cancer, FAK Box 6 expression is associated with increased tumor metastasis (Devaud et al., 2019). Thus, increased FAK expression associated with gene gains or alternatively spliced FAK isoforms can be drivers of tumor initiation and progression.

## Targeting FAK

### Small-molecule inhibitors targeting FAK or FAK–Pyk2 kinase activity

**Fig. 5.**
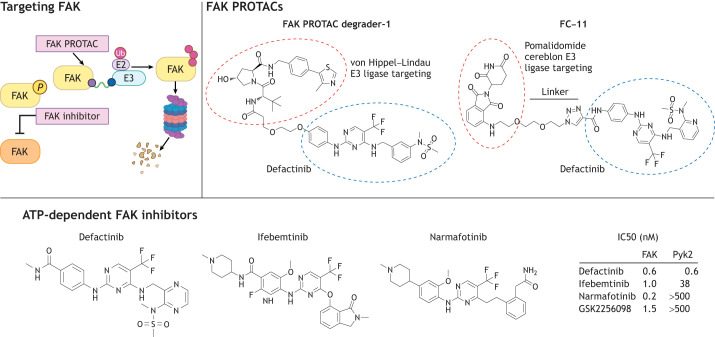
**Pharmacological approaches to FAK inhibition.** One approach to blocking FAK activity includes small molecules that are reversibly ATP competitive (left panel). The structures of defactinib, ifebemtinib and narmafotinib are shown (bottom). These compounds inhibit FAK activity and only defactinib inhibits Pyk2 in cells. Another approach is the use of proteolysis targeting chimera (PROTACs), which involves the chemical linkage of ATP-dependent FAK kinase inhibitors with E3 ligase recruitment moieties (von Hippel–Lindau or pomalidomide-cereblon binding), which target FAK for ubiquitylation (Ub) and proteasomal degradation (top right panel). Structures of FAK PROTAC Degrader-1 and FC-11 are shown; these comprise chemical linkages with defactinib (circled in blue) with different E3 ligase PROTAC moieties (circled in red). Clinical trials are in progress for several ATP-competitive FAK inhibitors, whereas FAK PROTACs are in preclinical development. Created using BioRender.com.

Several different small-molecule ATP-competitive inhibitors to FAK have been developed that exhibit high specificity, can be delivered orally (and in pill form to patients) and exhibit on-target FAK Y397 phosphorylation reduction in tumor and stromal cells (as reviewed by Spallarossa et al., 2022) (Fig. 5). As discussed above, increases in FAK Y397 phosphorylation might not always be reflective of ‘active FAK’. Nevertheless, loss of FAK Y397 phosphorylation parallels reduction in intrinsic FAK activity. FAK inhibitors are cell permeable and act to reduce normal and tumor adherent cell movement, but do not prevent cell proliferation at concentrations of 1 µM and below (Mitra et al., 2005; Slack-Davis et al., 2007).

It is important to note that some of the FAK inhibitors currently being tested in clinical trials exhibit >50-fold selectivity for FAK versus Pyk2. Ifebemtinib (InxMed Inc.) and narmafotinib (Amplia Therapeutics) are selective for FAK, whereas defactinib (Verastem Inc.) equally inhibits both FAK and Pyk2 kinase activities. Small-molecule Pyk2-specific inhibitors have been created, but not characterized in cells (Farand et al., 2016). Neither ifebemtinib nor defactinib prevent adherent tumor cell growth; however, both can negatively impact tumorsphere anchorage-independent cell proliferation and survival (Diaz Osterman et al., 2019; Laszlo et al., 2019; Tanjoni et al., 2010). Despite differences in targeting FAK or FAK–Pyk2, similar phenotypic findings have been observed to date with these inhibitors in cultured cell or mouse tumors (Dawson et al., 2021). In general, FAK and FAK–Pyk2 inhibitors are not cytotoxic drugs.

### Targeting FAK as a mechanotherapy

In addition to targeting tumors, the disruption of tensional signaling using small-molecule FAK inhibitors has revealed potential translational opportunities in promoting tissue regeneration and limiting scar formation. In mice, pharmacological FAK inhibition accelerates wound healing with reduced collagen deposition and fewer scar forming myofibroblasts (Ma et al., 2018). FAK inhibition also enhances skin graft healing in large mammals (Chen et al., 2021) with decreased fibrosis and contracture phenotypes (Chen et al., 2022). In atopic dermatitis, mechanical scratching is both causal for inflammatory skin ulceration, and associated with increased epidermal FAK Y397 phosphorylation (Jia et al., 2023). Pharmacological FAK inhibition delivered in a hydrogel dressing (also containing nanoparticle reactive-oxygen scavengers) reduces dermatitis-associated inflammation and epithelial barrier damage (Jia et al., 2023). Together, these studies highlight the potential clinical promise of inhibiting FAK as a mechano-therapy target.

### Targeting FAK for proteolysis

Recent advancements in chemical FAK inhibitor design have resulted in the generation of proteolysis targeting chimera (PROTAC) FAK degraders (Cromm et al., 2018). FAK PROTACs are chemical molecules designed to post-translationally target FAK by linking ATP-competitive FAK inhibitors with a chemical E3 ligase-binding moiety, such as pomalidomide, to recruit cereblon E3 ligase or a chemical structure to promote von Hippel–Lindau (VHL) E3 ligase binding (Fig. 5) (as reviewed by Chirnomas et al., 2023; Spallarossa et al., 2022). PROTACs are dependent on E3 ligase expression in target cells for target protein degradation and the chemical destruction of FAK should block both kinase and scaffolding signaling activities. In general, although early FAK PROTAC compound development identified molecules that inhibit tumor cell motility and invasion at nanomolar concentrations (Cromm et al., 2018), other FAK PROTAC compounds showed that FAK knockdown had no effect on tumor cell growth *in vitro* (Gao et al., 2020a; Popow et al., 2019). As FAK suppresses anoikis, this is best measured by anchorage-independent growth assays. Additionally, few of the published FAK PROTAC studies measured effects on Pyk2, which might function in parallel to FAK (as discussed above). Moreover, extended FAK PROTAC administration in mice has shown that these compounds might accumulate in tissues, with associated toxicity (Gao et al., 2020b).

However, recent studies have shown that FAK PROTAC addition to tumor cells *in vitro* at sub-micromolar concentrations recapitulates many of the FAK inhibitor-associated cell phenotypes (Hou et al., 2023; Law et al., 2021). Looking toward the future, FAK PROTAC modifications are still required to optimize oral delivery, cell penetration, pharmacokinetics and off-target toxicity (Koide et al., 2023). As genetic tumor models have revealed important differences resulting from FAK loss versus inactivation of FAK activity (Dawson et al., 2021), FAK PROTAC compounds hold great promise as tools to decipher consequences of FAK or FAK–Pyk2 inhibition versus loss of expression.

### Combining FAK inhibitors with other chemotherapies

FAK activation can occur downstream of oncogenes and in response to chemotherapy or environmental stress, which makes FAK an attractive target for several different cancers. In PDAC mouse models, oral FAK inhibitor administration enhances the cytotoxic effects of gemcitabine and increases responsiveness to combined anti-checkpoint receptor immunotherapy (Jiang et al., 2016; Osipov et al., 2021). FAK inhibition is also associated with anti-fibrotic effects on the pancreatic tumor stroma, changes that also enhance the effects of radiation therapy (Lander et al., 2022). Notably, in human PDAC tumors, FAK Y397 phosphorylation increases with disease progression and is greater in stromal compartments compared to tumor cells (Murphy et al., 2021; Zaghdoudi et al., 2020). In models of KRAS G12C oncogenic-driven non-small cell lung carcinoma, orally delivered FAK inhibitors radio-sensitize tumors (Tang et al., 2016), synergize with inhibitors of KRAS G12C (Zhang et al., 2021) and boost checkpoint inhibitor immune responses (Qiao et al., 2024) via tumor intrinsic and extrinsic mechanisms.

This type of intrinsic–extrinsic multi-factorial FAK inhibition has also been observed in ovarian cancer models whereby gains in FAK expression, FAK Y397 phosphorylation and FAK activity enhance intrinsic cisplatin and taxane resistance in part through transcriptomic changes (Diaz Osterman et al., 2019; Kang et al., 2013). In primary ovarian tumors, changes in extrinsic stromal matrix composition and tissue stiffness are associated with tumor FAK activation and cisplatin chemoresistance (Pietila et al., 2021). Moreover, tumor intrinsic FAK inhibition in mouse ovarian or SCC models results in enhanced T and B cell recruitment, potentiated activity of immune checkpoint antibodies and formation of tertiary lymphoid structures, which are known markers of immune activation (Canel et al., 2020; Ozmadenci et al., 2022; Serrels et al., 2015, 2017). In a Ras- and p53-mutated mouse PDAC model, loss of tumor FAK results in increased antigen processing and presentation that might impact immune responses (Canel et al., 2023). In summary, these studies show that FAK activation can impact (and be activated by) the tumor microenvironment and that FAK inhibition uncovers several tumor vulnerabilities altering immune recognition and cell survival.

## Ongoing FAK inhibitor clinical trials

Over the past 10 years, several early phase clinical trials have tested small-molecule FAK inhibitors from different companies (Dawson et al., 2021; Sulzmaier et al., 2014). These trials revealed that daily dosing for extended periods of time is safe and associated with low levels of manageable adverse events, and that small-molecule FAK inhibitors in general had good pharmacokinetic properties (de Jong et al., 2014; Jones et al., 2015; Wang-Gillam et al., 2022). However, in early phase II trials for malignant pleural mesothelioma and PDAC, the clinical endpoints were not met (Aung et al., 2022; Fennell et al., 2019). In unselected mesothelioma patients, defactinib (as maintenance after primary standard-of-care chemotherapy) was terminated early due to lack of efficacy (Fennell et al., 2019). Conversely, in a clinical trial of meningioma patients with somatic loss of the neurofibromatosis type II (*NF2*) gene, FAK inhibitor monotherapy extended progression-free survival at six months in grade 1–3 tumors compared to historical controls (Brastianos et al., 2022). This trial was testing the hypothesis that *NF2* loss creates a synthetic lethal relationship with FAK inhibition (Shapiro et al., 2014). Additionally, partial responses to defactinib monotherapy were reported in meningioma patients with *NF2* mutations as part of the NCI-MATCH trial (NCT04439331).

Despite limited effectiveness of defactinib monotherapy, several phase II trials are testing the role of defactinib FAK inhibition as part of combination chemotherapies (Table 1). The combination of defactinib with the RAF/MEK clamp avutometinib (VS-6766) was tested in patients with KRAS mutant lung or ovarian cancers (NCT03875820). Early phase II results in low-grade serous ovarian cancer (LGSOC) showed that a defactinib and avutometinib combination elicited an overall response rate (ORR) of 46% in 11 of 26 evaluable patients, which was 2–8-fold greater than the historical ORR in this pre-treated patient population.

**Table 1.**
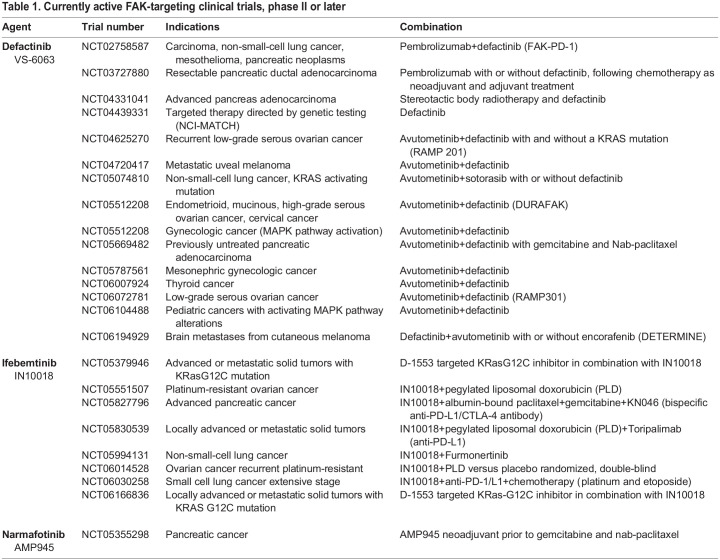
Currently active FAK-targeting clinical trials, phase II or later

LGSOC is a rare subtype that is histologically, molecularly and clinically distinct from HGSOC. Molecularly, LGSOC is characterized by chemoresistance, estrogen and progesterone receptor positivity, activation of the MAPK pathway and wild-type p53 expression (Cobb and Gershenson, 2023). Verastem was given ‘breakthrough therapy’ designation by the FDA in 2021 for the defactinib and avutometinib combination, which allows for expedited review of drugs for serious life-threatening conditions. Preliminary results for the Raf and MEK Program (RAMP201) phase II trial (NCT04625270) in LGSOC, testing the combination of defactinib and avutometinib, reveal an ORR of 45% (13/29) and tumor shrinkage in 86% of evaluable patients receiving combination treatment. A confirmatory international phase III (RAMP301, NCT06072781) trial has recently opened to recruitment, with patient enrollment coinciding with an expected application for FDA approval.

In 2019, InxMed Inc. purchased the rights to the FAK inhibitor BI853520 (from Boehringer Ingelheim) and renamed this compound ifebemtinib (InxMed, IN10018). A single arm phase I-II trial was subsequently started, testing ifebemtinib with pegylated doxorubicin (Doxil) in recurrent platinum-resistant HGSOC (NCT05551507) (Table 1), the most lethal gynecologic malignancy in the United States, with a historical response rate of less than 20%. As of April 2023, 54 evaluable patients showed one complete response, 21 partial responses, and 20 patients with stable disease for an ORR of 46% (Zhang et al., 2024). Currently, a randomized, double-blind phase II trial is evaluating ifebemtinib and Doxil versus placebo and Doxil in platinum-resistant recurrent HGSOC (NCT06014528).

## Conclusions

Cells are exposed to a variety of stimuli on a constant basis, including mitogenic and chemotactic factors, death signals and chemical insults, all delivered against a backdrop of various biomechanical alterations. In consideration of this complexity, it is believed that intermediary proteins function to contextualize and integrate discrete extracellular signals to elicit appropriate intracellular responses. FAK functions in this manner as a signal integration hub and as a master regulator of drug resistance. This involves FAK kinase-dependent and -independent signals, as well as FAK localization to focal adhesions, cell–cell contacts, endosomal membranes and in the nucleus. Herein, we have touched on how FAK plays an important signal integration role and ultimately functions to guide cellular behavior.

The ability of small-molecule FAK inhibitors to block kinase activity, yet permit scaffolding functions, might explain their relatively limited toxicity *in vivo*. The regulation of FAK activity represents an actionable target in a broad set of pathological conditions, ranging from tumors to stromal tissues, which includes targeting CAFs and ECs as well as altering tensional pathologies, such as wound healing. By contrast, FAK inhibitors have limited or possibly stimulatory effects on tumor-responsive immune cells. This range of targets, and limited toxicity of FAK inhibitors, might present a powerful tool to influence the physiological response to other therapeutic approaches. Indeed, after more than 30 years since the discovery of FAK, a clinical approval might finally be on the near-term horizon.

## References

[JCS261723C1] Acebron, I., Righetto, R. D., Schoenherr, C., de Buhr, S., Redondo, P., Culley, J., Rodriguez, C. F., Daday, C., Biyani, N., Llorca, O. et al. (2020). Structural basis of Focal Adhesion Kinase activation on lipid membranes. *EMBO J.* 39, e104743. 10.15252/embj.2020104743 32779739 PMC7527928

[JCS261723C2] Agochiya, M., Brunton, V. G., Owens, D. W., Parkinson, E. K., Paraskeva, C., Keith, W. N. and Frame, M. C. (1999). Increased dosage and amplification of the focal adhesion kinase gene in human cancer cells. *Oncogene* 18, 5646-5653. 10.1038/sj.onc.1202957 10523844

[JCS261723C3] Alanko, J. and Ivaska, J. (2016). Endosomes: emerging platforms for integrin-mediated FAK signalling. *Trends Cell Biol.* 26, 391-398. 10.1016/j.tcb.2016.02.001 26944773

[JCS261723C4] Aung, K. L., McWhirter, E., Welch, S., Wang, L., Lovell, S., Stayner, L. A., Ali, S., Malpage, A., Makepeace, B., Ramachandran, M. et al. (2022). A phase II trial of GSK2256098 and trametinib in patients with advanced pancreatic ductal adenocarcinoma. *J. Gastrointest. Oncol.* 13, 3216-3226. 10.21037/jgo-22-86 36636049 PMC9830369

[JCS261723C5] Bae, Y. H., Mui, K. L., Hsu, B. Y., Liu, S. L., Cretu, A., Razinia, Z., Xu, T., Pure, E. and Assoian, R. K. (2014). A FAK-Cas-Rac-lamellipodin signaling module transduces extracellular matrix stiffness into mechanosensitive cell cycling. *Sci. Signal.* 7, ra57. 24939893 10.1126/scisignal.2004838 PMC4345117

[JCS261723C6] Bauer, M. S., Baumann, F., Daday, C., Redondo, P., Durner, E., Jobst, M. A., Milles, L. F., Mercadante, D., Pippig, D. A., Gaub, H. E. et al. (2019). Structural and mechanistic insights into mechanoactivation of focal adhesion kinase. *Proc. Natl. Acad. Sci. USA* 116, 6766-6774. 10.1073/pnas.1820567116 30877242 PMC6452671

[JCS261723C7] Brastianos, P. K., Twohy, E. L., Gerstner, E. R., Kaufmann, T. J., Iafrate, A. J., Lennerz, J., Jeyapalan, S., Piccioni, D. E., Monga, V., Fadul, C. E. et al. (2022). Alliance A071401: phase II trial of focal adhesion kinase inhibition in meningiomas with somatic NF2 mutations. *J. Clin. Oncol.* 41, 618-628. 10.1200/JCO.21.02371 36288512 PMC9870228

[JCS261723C8] Burridge, K. (2017). Focal adhesions: a personal perspective on a half century of progress. *FEBS J.* 284, 3355-3361. 10.1111/febs.14195 28796323 PMC5643231

[JCS261723C9] Cai, X., Lietha, D., Ceccarelli, D. F., Karginov, A. V., Rajfur, Z., Jacobson, K., Hahn, K. M., Eck, M. J. and Schaller, M. D. (2008). Spatial and temporal regulation of focal adhesion kinase activity in living cells. *Mol. Cell. Biol.* 28, 201-214. 10.1128/MCB.01324-07 17967873 PMC2223290

[JCS261723C10] Canel, M., Byron, A., Sims, A. H., Cartier, J., Patel, H., Frame, M. C., Brunton, V. G., Serrels, B. and Serrels, A. (2017). Nuclear FAK and Runx1 cooperate to regulate IGFBP3, cell-cycle progression, and tumor growth. *Cancer Res.* 77, 5301-5312. 10.1158/0008-5472.CAN-17-0418 28807942 PMC6126615

[JCS261723C11] Canel, M., Taggart, D., Sims, A. H., Lonergan, D. W., Waizenegger, I. C. and Serrels, A. (2020). T-cell co-stimulation in combination with targeting FAK drives enhanced anti-tumor immunity. *Elife* 9, e48092. 10.7554/eLife.48092 31959281 PMC6974352

[JCS261723C12] Canel, M., Slawinska, A. D., Lonergan, D. W., Kallor, A. A., Upstill-Goddard, R., Davidson, C., von Kriegsheim, A., Biankin, A. V., Byron, A., Alfaro, J. et al. (2023). FAK suppresses antigen processing and presentation to promote immune evasion in pancreatic cancer. *Gut* 73, 131-155. 10.1136/gutjnl-2022-327927 36977556 PMC10715489

[JCS261723C13] Carisey, A., Tsang, R., Greiner, A. M., Nijenhuis, N., Heath, N., Nazgiewicz, A., Kemkemer, R., Derby, B., Spatz, J. and Ballestrem, C. (2013). Vinculin regulates the recruitment and release of core focal adhesion proteins in a force-dependent manner. *Curr. Biol.* 23, 271-281. 10.1016/j.cub.2013.01.009 23375895 PMC3580286

[JCS261723C14] Chan, K. T., Cortesio, C. L. and Huttenlocher, A. (2009). FAK alters invadopodia and focal adhesion composition and dynamics to regulate breast cancer invasion. *J. Cell Biol.* 185, 357-370. 10.1083/jcb.200809110 19364917 PMC2700377

[JCS261723C15] Chen, X. L., Nam, J. O., Jean, C., Lawson, C., Walsh, C. T., Goka, E., Lim, S. T., Tomar, A., Tancioni, I., Uryu, S. et al. (2012). VEGF-induced vascular permeability is mediated by FAK. *Dev. Cell* 22, 146-157. 10.1016/j.devcel.2011.11.002 22264731 PMC3266538

[JCS261723C16] Chen, G., Gao, C., Gao, X., Zhang, D. H., Kuan, S. F., Burns, T. F. and Hu, J. (2018). Wnt/beta-catenin pathway activation mediates adaptive resistance to BRAF inhibition in colorectal cancer. *Mol. Cancer Ther.* 17, 806-813. 10.1158/1535-7163.MCT-17-0561 29167314 PMC5882543

[JCS261723C17] Chen, K., Kwon, S. H., Henn, D., Kuehlmann, B. A., Tevlin, R., Bonham, C. A., Griffin, M., Trotsyuk, A. A., Borrelli, M. R., Noishiki, C. et al. (2021). Disrupting biological sensors of force promotes tissue regeneration in large organisms. *Nat. Commun.* 12, 5256. 10.1038/s41467-021-25410-z 34489407 PMC8421385

[JCS261723C18] Chen, K., Henn, D., Januszyk, M., Barrera, J. A., Noishiki, C., Bonham, C. A., Griffin, M., Tevlin, R., Carlomagno, T., Shannon, T. et al. (2022). Disrupting mechanotransduction decreases fibrosis and contracture in split-thickness skin grafting. *Sci. Transl. Med.* 14, eabj9152. 10.1126/scitranslmed.abj9152 35584231

[JCS261723C19] Chirnomas, D., Hornberger, K. R. and Crews, C. M. (2023). Protein degraders enter the clinic - a new approach to cancer therapy. *Nat. Rev. Clin. Oncol.* 20, 265-278. 10.1038/s41571-023-00736-3 36781982 PMC11698446

[JCS261723C20] Choi, C. H., Webb, B. A., Chimenti, M. S., Jacobson, M. P. and Barber, D. L. (2013). pH sensing by FAK-His58 regulates focal adhesion remodeling. *J. Cell Biol.* 202, 849-859. 10.1083/jcb.201302131 24043700 PMC3776353

[JCS261723C21] Cobb, L. and Gershenson, D. (2023). Novel therapeutics in low-grade serous ovarian cancer. *Int. J. Gynecol. Cancer* 33, 377-384. 10.1136/ijgc-2022-003677 36878564

[JCS261723C22] Cohen, L. A. and Guan, J. L. (2005). Residues within the first subdomain of the FERM-like domain in focal adhesion kinase are important in its regulation. *J. Biol. Chem.* 280, 8197-8207. 10.1074/jbc.M412021200 15611137

[JCS261723C23] Cooper, J. and Giancotti, F. G. (2019). Integrin signaling in cancer: mechanotransduction, stemness, epithelial plasticity, and therapeutic resistance. *Cancer Cell* 35, 347-367. 10.1016/j.ccell.2019.01.007 30889378 PMC6684107

[JCS261723C24] Cromm, P. M., Samarasinghe, K. T. G., Hines, J. and Crews, C. M. (2018). Addressing kinase-independent functions of Fak via PROTAC-mediated degradation. *J. Am. Chem. Soc.* 140, 17019-17026. 10.1021/jacs.8b08008 30444612

[JCS261723C25] Dawson, J. C., Serrels, A., Stupack, D. G., Schlaepfer, D. D. and Frame, M. C. (2021). Targeting FAK in anticancer combination therapies. *Nat. Rev. Cancer* 21, 313-324. 10.1038/s41568-021-00340-6 33731845 PMC8276817

[JCS261723C26] de Jong, M., Essers, J. and van Weerden, W. M. (2014). Imaging preclinical tumour models: improving translational power. *Nat. Rev. Cancer* 14, 481-493. 10.1038/nrc3751 24943811

[JCS261723C27] Devaud, C., Tilkin-Mariame, A. F., Vignolle-Vidoni, A., Souleres, P., Denadai-Souza, A., Rolland, C., Duthoit, C., Blanpied, C., Chabot, S., Bouille, P. et al. (2019). FAK alternative splice mRNA variants expression pattern in colorectal cancer. *Int. J. Cancer* 145, 494-502. 10.1002/ijc.32120 30628725 PMC6563491

[JCS261723C28] Diaz Osterman, C. J., Ozmadenci, D., Kleinschmidt, E. G., Taylor, K. N., Barrie, A. M., Jiang, S., Bean, L. M., Sulzmaier, F. J., Jean, C., Tancioni, I. et al. (2019). FAK activity sustains intrinsic and acquired ovarian cancer resistance to platinum chemotherapy. *Elife* 8, e47327. 10.7554/eLife.47327 31478830 PMC6721800

[JCS261723C29] Farand, J., Mai, N., Chandrasekhar, J., Newby, Z. E., Van Veldhuizen, J., Loyer-Drew, J., Venkataramani, C., Guerrero, J., Kwok, A., Li, N. et al. (2016). Selectivity switch between FAK and Pyk2: Macrocyclization of FAK inhibitors improves Pyk2 potency. *Bioorg. Med. Chem. Lett.* 26, 5926-5930. 10.1016/j.bmcl.2016.10.092 27876318

[JCS261723C30] Fatherree, J. P., Guarin, J. R., McGinn, R. A., Naber, S. P. and Oudin, M. J. (2022). Chemotherapy-induced collagen IV drives cancer cell motility through activation of Src and focal adhesion kinase. *Cancer Res.* 82, 2031-2044. 10.1158/0008-5472.CAN-21-1823 35260882 PMC9381104

[JCS261723C31] Fennell, D. A., Baas, P., Taylor, P., Nowak, A. K., Gilligan, D., Nakano, T., Pachter, J. A., Weaver, D. T., Scherpereel, A., Pavlakis, N. et al. (2019). Maintenance defactinib versus placebo after first-line chemotherapy in patients with merlin-stratified pleural mesothelioma: COMMAND-a double-blind, randomized, phase II study. *J. Clin. Oncol.* 37, 790-798. 10.1200/JCO.2018.79.0543 30785827

[JCS261723C32] Frame, M. C., Patel, H., Serrels, B., Lietha, D. and Eck, M. J. (2010). The FERM domain: organizing the structure and function of FAK. *Nat. Rev. Mol. Cell Biol.* 11, 802-814. 10.1038/nrm2996 20966971

[JCS261723C33] Frisch, S. M., Vuori, K., Ruoslahti, E. and Chan-Hui, P. Y. (1996). Control of adhesion-dependent cell survival by focal adhesion kinase. *J. Cell Biol.* 134, 793-799. 10.1083/jcb.134.3.793 8707856 PMC2120934

[JCS261723C34] Gao, C., Chen, G., Kuan, S. F., Zhang, D. H., Schlaepfer, D. D. and Hu, J. (2015). FAK/PYK2 promotes the Wnt/beta-catenin pathway and intestinal tumorigenesis by phosphorylating GSK3beta. *Elife* 4, e10072. 10.7554/eLife.10072 26274564 PMC4558782

[JCS261723C35] Gao, H., Wu, Y., Sun, Y., Yang, Y., Zhou, G. and Rao, Y. (2020a). Design, synthesis, and evaluation of highly potent FAK-targeting PROTACs. *ACS Med. Chem. Lett.* 11, 1855-1862. 10.1021/acsmedchemlett.9b00372 33062164 PMC7549110

[JCS261723C36] Gao, H., Zheng, C., Du, J., Wu, Y., Sun, Y., Han, C., Kee, K. and Rao, Y. (2020b). FAK-targeting PROTAC as a chemical tool for the investigation of non-enzymatic FAK function in mice. *Protein Cell* 11, 534-539. 10.1007/s13238-020-00732-8 32451721 PMC7305269

[JCS261723C37] Genna, A. and Gil-Henn, H. (2018). FAK family kinases: The Yin and Yang of cancer cell invasiveness. *Mol. Cell Oncol.* 5, e1449584. 10.1080/23723556.2018.1449584 30250911 PMC6149990

[JCS261723C38] Genna, A., Lapetina, S., Lukic, N., Twafra, S., Meirson, T., Sharma, V. P., Condeelis, J. S. and Gil-Henn, H. (2018). Pyk2 and FAK differentially regulate invadopodia formation and function in breast cancer cells. *J. Cell Biol.* 217, 375-395. 10.1083/jcb.201702184 29133485 PMC5748976

[JCS261723C39] Gil-Henn, H., Girault, J. A. and Lev, S. (2024). PYK2, a hub of signaling networks in breast cancer progression. *Trends Cell Biol*. 34, 312-326. 10.1016/j.tcb.2023.07.006 37586982

[JCS261723C40] Goni, G. M., Epifano, C., Boskovic, J., Camacho-Artacho, M., Zhou, J., Bronowska, A., Martin, M. T., Eck, M. J., Kremer, L., Grater, F. et al. (2014). Phosphatidylinositol 4,5-bisphosphate triggers activation of focal adhesion kinase by inducing clustering and conformational changes. *Proc. Natl. Acad. Sci. USA* 111, E3177-E3186. 25049397 10.1073/pnas.1317022111 PMC4128148

[JCS261723C41] Goode, E. L., Chenevix-Trench, G., Song, H., Ramus, S. J., Notaridou, M., Lawrenson, K., Widschwendter, M., Vierkant, R. A., Larson, M. C., Kjaer, S. K. et al. (2010). A genome-wide association study identifies susceptibility loci for ovarian cancer at 2q31 and 8q24. *Nat. Genet.* 42, 874-879. 10.1038/ng.668 20852632 PMC3020231

[JCS261723C42] Gorringe, K. L., George, J., Anglesio, M. S., Ramakrishna, M., Etemadmoghadam, D., Cowin, P., Sridhar, A., Williams, L. H., Boyle, S. E., Yanaihara, N. et al. (2010). Copy number analysis identifies novel interactions between genomic loci in ovarian cancer. *PLoS One* 5, e11408. 10.1371/journal.pone.0011408 20844748 PMC2937017

[JCS261723C43] Grashoff, C., Hoffman, B. D., Brenner, M. D., Zhou, R., Parsons, M., Yang, M. T., McLean, M. A., Sligar, S. G., Chen, C. S., Ha, T. et al. (2010). Measuring mechanical tension across vinculin reveals regulation of focal adhesion dynamics. *Nature* 466, 263-266. 10.1038/nature09198 20613844 PMC2901888

[JCS261723C44] Griffith, B. G. C., Upstill-Goddard, R., Brunton, H., Grimes, G. R., Biankin, A. V., Serrels, B., Byron, A. and Frame, M. C. (2021). FAK regulates IL-33 expression by controlling chromatin accessibility at c-Jun motifs. *Sci. Rep.* 11, 229. 10.1038/s41598-020-80111-9 33420223 PMC7794255

[JCS261723C45] Hamidi, H. and Ivaska, J. (2018). Every step of the way: integrins in cancer progression and metastasis. *Nat. Rev. Cancer* 18, 533-548. 10.1038/s41568-018-0038-z 30002479 PMC6629548

[JCS261723C46] Hanahan, D. and Weinberg, R. A. (2000). The hallmarks of cancer. *Cell* 100, 57-70. 10.1016/S0092-8674(00)81683-9 10647931

[JCS261723C47] Heim, J. B., McDonald, C. A., Wyles, S. P., Sominidi-Damodaran, S., Squirewell, E. J., Li, M., Motsonelidze, C., Bottcher, R. T., van Deursen, J. and Meves, A. (2018). FAK auto-phosphorylation site tyrosine 397 is required for development but dispensable for normal skin homeostasis. *PLoS One* 13, e0200558. 30001432 10.1371/journal.pone.0200558 PMC6042779

[JCS261723C48] Hirata, E., Girotti, M. R., Viros, A., Hooper, S., Spencer-Dene, B., Matsuda, M., Larkin, J., Marais, R. and Sahai, E. (2015). Intravital imaging reveals how BRAF inhibition generates drug-tolerant microenvironments with high integrin beta1/FAK signaling. *Cancer Cell* 27, 574-588. 10.1016/j.ccell.2015.03.008 25873177 PMC4402404

[JCS261723C49] Holland, E. N., Fernandez-Yague, M. A., Zhou, D. W., O'Neill, E. B., Woodfolk, A. U., Mora-Boza, A., Fu, J., Schlaepfer, D. D. and Garcia, A. J. (2024). FAK, vinculin, and talin control mechanosensitive YAP nuclear localization. *Biomaterials* 308, 122542. 10.1016/j.biomaterials.2024.122542 38547833 PMC11065566

[JCS261723C50] Hou, W., Gad, S. A., Ding, X., Dhanarajan, A. and Qiu, W. (2023). Focal adhesion kinase confers lenvatinib resistance in hepatocellular carcinoma via the regulation of lysine-deficient kinase 1. *Mol. Carcinog.* 63, 173-189. 10.1002/mc.23644 37787401 PMC10842616

[JCS261723C51] Hsia, D. A., Mitra, S. K., Hauck, C. R., Streblow, D. N., Nelson, J. A., Ilic, D., Huang, S., Li, E., Nemerow, G. R., Leng, J. et al. (2003). Differential regulation of cell motility and invasion by FAK. *J. Cell Biol.* 160, 753-767. 10.1083/jcb.200212114 12615911 PMC2173366

[JCS261723C52] Ilic, D., Furuta, Y., Kanazawa, S., Takeda, N., Sobue, K., Nakatsuji, N., Nomura, S., Fujimoto, J., Okada, M. and Yamamoto, T. (1995). Reduced cell motility and enhanced focal adhesion contact formation in cells from FAK-deficient mice. *Nature* 377, 539-544. 10.1038/377539a0 7566154

[JCS261723C53] Jean, C., Chen, X. L., Nam, J. O., Tancioni, I., Uryu, S., Lawson, C., Ward, K. K., Walsh, C. T., Miller, N. L., Ghassemian, M. et al. (2014). Inhibition of endothelial FAK activity prevents tumor metastasis by enhancing barrier function. *J. Cell Biol.* 204, 247-263. 10.1083/jcb.201307067 24446483 PMC3897185

[JCS261723C54] Jeong, K., Kim, J. H., Murphy, J. M., Park, H., Kim, S. J., Rodriguez, Y. A. R., Kong, H., Choi, C., Guan, J. L., Taylor, J. M. et al. (2019). Nuclear focal adhesion kinase controls vascular smooth muscle cell proliferation and neointimal hyperplasia through GATA4-mediated Cyclin D1 transcription. *Circ. Res.* 125, 152-166. 10.1161/CIRCRESAHA.118.314344 31096851 PMC6702425

[JCS261723C55] Jeong, K., Murphy, J. M., Ahn, E. E. and Lim, S. S. (2022). FAK in the nucleus prevents VSMC proliferation by promoting p27 and p21 expression via Skp2 degradation. *Cardiovasc. Res.* 118, 1150-1163. 10.1093/cvr/cvab132 33839758 PMC8930076

[JCS261723C56] Jeong, K., Murphy, J. M., Kim, J. H., Campbell, P. M., Park, H., Rodriguez, Y. A. R., Choi, C. S., Kim, J. S., Park, S., Kim, H. J. et al. (2021). FAK activation promotes SMC dedifferentiation via increased DNA methylation in contractile genes. *Circ. Res.* 129, e215-e233. 10.1161/CIRCRESAHA.121.319066 34702049 PMC8639767

[JCS261723C57] Jia, Y., Hu, J., An, K., Zhao, Q., Dang, Y., Liu, H., Wei, Z., Geng, S. and Xu, F. (2023). Hydrogel dressing integrating FAK inhibition and ROS scavenging for mechano-chemical treatment of atopic dermatitis. *Nat. Commun.* 14, 2478. 10.1038/s41467-023-38209-x 37120459 PMC10148840

[JCS261723C58] Jiang, H., Hegde, S., Knolhoff, B. L., Zhu, Y., Herndon, J. M., Meyer, M. A., Nywening, T. M., Hawkins, W. G., Shapiro, I. M., Weaver, D. T. et al. (2016). Targeting focal adhesion kinase renders pancreatic cancers responsive to checkpoint immunotherapy. *Nat. Med.* 22, 851-860. 10.1038/nm.4123 27376576 PMC4935930

[JCS261723C59] Jiang, H., Liu, X., Knolhoff, B. L., Hegde, S., Lee, K. B., Jiang, H., Fields, R. C., Pachter, J. A., Lim, K. H. and DeNardo, D. G. (2020). Development of resistance to FAK inhibition in pancreatic cancer is linked to stromal depletion. *Gut* 69, 122-132. 10.1136/gutjnl-2018-317424 31076405 PMC7167297

[JCS261723C60] Jones, S. F., Siu, L. L., Bendell, J. C., Cleary, J. M., Razak, A. R., Infante, J. R., Pandya, S. S., Bedard, P. L., Pierce, K. J., Houk, B. et al. (2015). A phase I study of VS-6063, a second-generation focal adhesion kinase inhibitor, in patients with advanced solid tumors. *Invest. New Drugs* 33, 1100-1107. 10.1007/s10637-015-0282-y 26334219

[JCS261723C61] Kadare, G., Gervasi, N., Brami-Cherrier, K., Blockus, H., El Messari, S., Arold, S. T. and Girault, J. A. (2015). Conformational dynamics of the focal adhesion targeting domain control specific functions of focal adhesion kinase in cells. *J. Biol. Chem.* 290, 478-491. 10.1074/jbc.M114.593632 25391654 PMC4281750

[JCS261723C62] Kang, Y., Hu, W., Ivan, C., Dalton, H. J., Miyake, T., Pecot, C. V., Zand, B., Liu, T., Huang, J., Jennings, N. B. et al. (2013). Role of focal adhesion kinase in regulating YB-1-mediated paclitaxel resistance in ovarian cancer. *J. Natl. Cancer Inst.* 105, 1485-1495. 10.1093/jnci/djt210 24062525 PMC3787907

[JCS261723C63] Kaveh, F., Baumbusch, L. O., Nebdal, D., Borresen-Dale, A. L., Lingjaerde, O. C., Edvardsen, H., Kristensen, V. N. and Solvang, H. K. (2016). A systematic comparison of copy number alterations in four types of female cancer. *BMC Cancer* 16, 913. 10.1186/s12885-016-2899-4 27876019 PMC5120489

[JCS261723C64] Kessler, B. E., Mishall, K. M., Kellett, M. D., Clark, E. G., Pugazhenthi, U., Pozdeyev, N., Kim, J., Tan, A. C. and Schweppe, R. E. (2019). Resistance to Src inhibition alters the BRAF-mutant tumor secretome to promote an invasive phenotype and therapeutic escape through a FAK>p130Cas>c-Jun signaling axis. *Oncogene* 38, 2565-2579. 10.1038/s41388-018-0617-1 30531837 PMC6450711

[JCS261723C65] Kim, L. C., Song, L. and Haura, E. B. (2009). Src kinases as therapeutic targets for cancer. *Nat. Rev. Clin. Oncol.* 6, 587-595. 10.1038/nrclinonc.2009.129 19787002

[JCS261723C66] Klingbeil, C. K., Hauck, C. R., Hsia, D. A., Jones, K. C., Reider, S. R. and Schlaepfer, D. D. (2001). Targeting Pyk2 to beta 1-integrin-containing focal contacts rescues fibronectin-stimulated signaling and haptotactic motility defects of focal adhesion kinase-null cells. *J. Cell Biol.* 152, 97-110. 10.1083/jcb.152.1.97 11149924 PMC2193658

[JCS261723C67] Koide, E., Mohardt, M. L., Doctor, Z. M., Yang, A., Hao, M., Donovan, K. A., Kuismi, C. C., Nelson, A. J., Abell, K., Aguiar, M. et al. (2023). Development and characterization of selective FAK inhibitors and PROTACs with in vivo activity. *Chembiochem* 24, e202300141. 37088717 10.1002/cbic.202300141 PMC10590827

[JCS261723C68] Kolev, V. N., Tam, W. F., Wright, Q. G., McDermott, S. P., Vidal, C. M., Shapiro, I. M., Xu, Q., Wicha, M. S., Pachter, J. A. and Weaver, D. T. (2017). Inhibition of FAK kinase activity preferentially targets cancer stem cells. *Oncotarget* 8, 51733-51747. 10.18632/oncotarget.18517 28881682 PMC5584283

[JCS261723C69] Lander, V. E., Belle, J. I., Kingston, N. L., Herndon, J. M., Hogg, G. D., Liu, X., Kang, L. I., Knolhoff, B. L., Bogner, S. J., Baer, J. M. et al. (2022). Stromal reprogramming by FAK inhibition overcomes radiation resistance to allow for immune priming and response to checkpoint blockade. *Cancer Discov.* 12, 2774-2799. 10.1158/2159-8290.CD-22-0192 36165893 PMC9722639

[JCS261723C70] Laszlo, V., Valko, Z., Ozsvar, J., Kovacs, I., Garay, T., Hoda, M. A., Klikovits, T., Stockhammer, P., Aigner, C., Groger, M. et al. (2019). The FAK inhibitor BI 853520 inhibits spheroid formation and orthotopic tumor growth in malignant pleural mesothelioma. *J. Mol. Med. (Berl)* 97, 231-242. 10.1007/s00109-018-1725-7 30539198 PMC6348072

[JCS261723C71] Law, R. P., Nunes, J., Chung, C. W., Bantscheff, M., Buda, K., Dai, H., Evans, J. P., Flinders, A., Klimaszewska, D., Lewis, A. J. et al. (2021). Discovery and Characterisation of Highly Cooperative FAK-Degrading PROTACs. *Angew. Chem. Int. Ed. Engl.* 60, 23327-23334. 10.1002/anie.202109237 34416073

[JCS261723C72] Lawson, C., Lim, S. T., Uryu, S., Chen, X. L., Calderwood, D. A. and Schlaepfer, D. D. (2012). FAK promotes recruitment of talin to nascent adhesions to control cell motility. *J. Cell Biol.* 196, 223-232. 10.1083/jcb.201108078 22270917 PMC3265949

[JCS261723C73] Le Coq, J., Acebron, I., Rodrigo Martin, B., Lopez Navajas, P. and Lietha, D. (2022). New insights into FAK structure and function in focal adhesions. *J. Cell Sci.* 135, jcs.259089. 10.1242/jcs.259089 36239192

[JCS261723C74] Levental, K. R., Yu, H., Kass, L., Lakins, J. N., Egeblad, M., Erler, J. T., Fong, S. F., Csiszar, K., Giaccia, A., Weninger, W. et al. (2009). Matrix crosslinking forces tumor progression by enhancing integrin signaling. *Cell* 139, 891-906. 10.1016/j.cell.2009.10.027 19931152 PMC2788004

[JCS261723C75] Li, X., Combs, J. D., III, Salaita, K. and Shu, X. (2023). Polarized focal adhesion kinase activity within a focal adhesion during cell migration. *Nat. Chem. Biol.* 19, 1458-1468. 10.1038/s41589-023-01353-y 37349581 PMC10732478

[JCS261723C76] Lietha, D., Cai, X., Ceccarelli, D. F., Li, Y., Schaller, M. D. and Eck, M. J. (2007). Structural basis for the autoinhibition of focal adhesion kinase. *Cell* 129, 1177-1187. 10.1016/j.cell.2007.05.041 17574028 PMC2077847

[JCS261723C77] Lim, S. T., Chen, X. L., Lim, Y., Hanson, D. A., Vo, T. T., Howerton, K., Larocque, N., Fisher, S. J., Schlaepfer, D. D. and Ilic, D. (2008). Nuclear FAK promotes cell proliferation and survival through FERM-enhanced p53 degradation. *Mol. Cell* 29, 9-22. 10.1016/j.molcel.2007.11.031 18206965 PMC2234035

[JCS261723C78] Lim, S. T., Chen, X. L., Tomar, A., Miller, N. L., Yoo, J. and Schlaepfer, D. D. (2010a). Knock-in mutation reveals an essential role for focal adhesion kinase activity in blood vessel morphogenesis and cell motility-polarity but not cell proliferation. *J. Biol. Chem.* 285, 21526-21536. 10.1074/jbc.M110.129999 20442405 PMC2898428

[JCS261723C79] Lim, S. T., Miller, N. L., Nam, J. O., Chen, X. L., Lim, Y. and Schlaepfer, D. D. (2010b). Pyk2 inhibition of p53 as an adaptive and intrinsic mechanism facilitating cell proliferation and survival. *J. Biol. Chem.* 285, 1743-1753. 10.1074/jbc.M109.064212 19880522 PMC2804332

[JCS261723C80] Lim, S. T., Miller, N. L., Chen, X. L., Tancioni, I., Walsh, C. T., Lawson, C., Uryu, S., Weis, S. M., Cheresh, D. A. and Schlaepfer, D. D. (2012). Nuclear-localized focal adhesion kinase regulates inflammatory VCAM-1 expression. *J. Cell Biol.* 197, 907-919. 10.1083/jcb.201109067 22734001 PMC3384409

[JCS261723C81] Ma, K., Kwon, S. H., Padmanabhan, J., Duscher, D., Trotsyuk, A. A., Dong, Y., Inayathullah, M., Rajadas, J. and Gurtner, G. C. (2018). Controlled delivery of a focal adhesion kinase inhibitor results in accelerated wound closure with decreased scar formation. *J. Invest. Dermatol.* 138, 2452-2460. 10.1016/j.jid.2018.04.034 29775632

[JCS261723C82] Maniati, E., Berlato, C., Gopinathan, G., Heath, O., Kotantaki, P., Lakhani, A., McDermott, J., Pegrum, C., Delaine-Smith, R. M., Pearce, O. M. T. et al. (2020). Mouse ovarian cancer models recapitulate the human tumor microenvironment and patient response to treatment. *Cell Rep.* 30, 525-540.e7. 10.1016/j.celrep.2019.12.034 31940494 PMC6963791

[JCS261723C83] Marlowe, T., Dementiev, A., Figel, S., Rivera, A., Flavin, M. and Cance, W. (2019). High resolution crystal structure of the FAK FERM domain reveals new insights on the Druggability of tyrosine 397 and the Src SH3 binding site. *BMC Mol. Cell Biol.* 20, 10. 10.1186/s12860-019-0193-4 31109284 PMC6528292

[JCS261723C84] McLean, G. W., Carragher, N. O., Avizienyte, E., Evans, J., Brunton, V. G. and Frame, M. C. (2005). The role of focal-adhesion kinase in cancer - a new therapeutic opportunity. *Nat. Rev. Cancer* 5, 505-515. 10.1038/nrc1647 16069815

[JCS261723C85] Mierke, C. T., Fischer, T., Puder, S., Kunschmann, T., Soetje, B. and Ziegler, W. H. (2017). Focal adhesion kinase activity is required for actomyosin contractility-based invasion of cells into dense 3D matrices. *Sci. Rep.* 7, 42780. 10.1038/srep42780 28202937 PMC5311912

[JCS261723C86] Mitra, S. K. and Schlaepfer, D. D. (2006). Integrin-regulated FAK-Src signaling in normal and cancer cells. *Curr. Opin. Cell Biol.* 18, 516-523. 10.1016/j.ceb.2006.08.011 16919435

[JCS261723C87] Mitra, S. K., Hanson, D. A. and Schlaepfer, D. D. (2005). Focal adhesion kinase: in command and control of cell motility. *Nat. Rev. Mol. Cell Biol.* 6, 56-68. 10.1038/nrm1549 15688067

[JCS261723C88] Mitra, S. K., Lim, S. T., Chi, A. and Schlaepfer, D. D. (2006a). Intrinsic focal adhesion kinase activity controls orthotopic breast carcinoma metastasis via the regulation of urokinase plasminogen activator expression in a syngeneic tumor model. *Oncogene* 25, 4429-4440. 10.1038/sj.onc.1209482 16547501

[JCS261723C89] Mitra, S. K., Mikolon, D., Molina, J. E., Hsia, D. A., Hanson, D. A., Chi, A., Lim, S. T., Bernard-Trifilo, J. A., Ilic, D., Stupack, D. G. et al. (2006b). Intrinsic FAK activity and Y925 phosphorylation facilitate an angiogenic switch in tumors. *Oncogene* 25, 5969-5984. 10.1038/sj.onc.1209588 16682956

[JCS261723C90] Mousson, A., Legrand, M., Steffan, T., Vauchelles, R., Carl, P., Gies, J. P., Lehmann, M., Zuber, G., De Mey, J., Dujardin, D. et al. (2021). Inhibiting FAK-paxillin interaction reduces migration and invadopodia-mediated matrix degradation in metastatic melanoma cells. *Cancers (Basel)* 13, 1871. 10.3390/cancers13081871 33919725 PMC8070677

[JCS261723C91] Murphy, K. J., Reed, D. A., Vennin, C., Conway, J. R. W., Nobis, M., Yin, J. X., Chambers, C. R., Pereira, B. A., Lee, V., Filipe, E. C. et al. (2021). Intravital imaging technology guides FAK-mediated priming in pancreatic cancer precision medicine according to Merlin status. *Sci. Adv.* 7, eabh0363. 10.1126/sciadv.abh0363 34586840 PMC8480933

[JCS261723C92] Murphy, J. M., Jeong, K., Tran, D. T. K., Cioffi, D. L., Campbell, P. M., Kim, J. H., Jo, H., Ahn, E. E. and Lim, S. S. (2023). Nuclear FAK in endothelium: An intrinsic inhibitor of NF-kappaB activation in atherosclerosis. *Atherosclerosis* 379, 117189. 10.1016/j.atherosclerosis.2023.117189 37527611 PMC10530536

[JCS261723C93] Newport, E., Pedrosa, A. R., Lees, D., Dukinfield, M., Carter, E., Gomez-Escudero, J., Casado, P., Rajeeve, V., Reynolds, L. E., R, P. (2022). Elucidating the role of the kinase activity of endothelial cell focal adhesion kinase in angiocrine signalling and tumour growth. *J. Pathol.* 256, 235-247. 10.1002/path.5833 34743335

[JCS261723C94] Osipov, A., Blair, A. B., Liberto, J., Wang, J., Li, K., Herbst, B., Xu, Y., Li, S., Niu, N., Rashid, R. et al. (2021). Inhibition of focal adhesion kinase enhances antitumor response of radiation therapy in pancreatic cancer through CD8+ T cells. *Cancer Biol. Med.* 18, 206-214. 10.20892/j.issn.2095-3941.2020.0273 33628595 PMC7877172

[JCS261723C95] Ozmadenci, D., Shankara Narayanan, J. S., Andrew, J., Ojalill, M., Barrie, A. M., Jiang, S., Iyer, S., Chen, X. L., Rose, M., Estrada, V. et al. (2022). Tumor FAK orchestrates immunosuppression in ovarian cancer via the CD155/TIGIT axis. *Proc. Natl. Acad. Sci. USA* 119, e2117065119. 10.1073/pnas.2117065119 35467979 PMC9169934

[JCS261723C96] Pang, X., He, X., Qiu, Z., Zhang, H., Xie, R., Liu, Z., Gu, Y., Zhao, N., Xiang, Q. and Cui, Y. (2023). Targeting integrin pathways: mechanisms and advances in therapy. *Signal. Transduct. Target Ther.* 8, 1. 10.1038/s41392-022-01259-6 36588107 PMC9805914

[JCS261723C97] Paracchini, L., Beltrame, L., Grassi, T., Inglesi, A., Fruscio, R., Landoni, F., Ippolito, D., Delle Marchette, M., Paderno, M., Adorni, M. et al. (2021). Genome-wide copy-number alterations in circulating tumor DNA as a novel biomarker for patients with high-grade serous ovarian cancer. *Clin. Cancer Res.* 27, 2549-2559. 10.1158/1078-0432.CCR-20-3345 33323403

[JCS261723C98] Parsons, J. T. (2003). Focal adhesion kinase: the first ten years. *J. Cell Sci.* 116, 1409-1416. 10.1242/jcs.00373 12640026

[JCS261723C99] Pedrosa, A. R., Bodrug, N., Gomez-Escudero, J., Carter, E. P., Reynolds, L. E., Georgiou, P. N., Fernandez, I., Lees, D. M., Kostourou, V., Alexopoulou, A. N. et al. (2019). Tumor angiogenesis is differentially regulated by phosphorylation of endothelial cell focal adhesion kinase tyrosines-397 and -861. *Cancer Res.* 79, 4371-4386. 10.1158/0008-5472.CAN-18-3934 31189647

[JCS261723C100] Pietila, E. A., Gonzalez-Molina, J., Moyano-Galceran, L., Jamalzadeh, S., Zhang, K., Lehtinen, L., Turunen, S. P., Martins, T. A., Gultekin, O., Lamminen, T. et al. (2021). Co-evolution of matrisome and adaptive adhesion dynamics drives ovarian cancer chemoresistance. *Nat. Commun.* 12, 3904. 10.1038/s41467-021-24009-8 34162871 PMC8222388

[JCS261723C101] Pifer, P. M., Yang, L., Kumar, M., Xie, T., Frederick, M., Hefner, A., Beadle, B., Molkentine, D., Molkentine, J., Dhawan, A. et al. (2023). FAK drives resistance to therapy in HPV-negative head and neck cancer in a p53-dependent manner. *Clin. Cancer Res.* 30, 187-197. 10.1158/1078-0432.CCR-23-0964 PMC10767302 37819945

[JCS261723C102] Popow, J., Arnhof, H., Bader, G., Berger, H., Ciulli, A., Covini, D., Dank, C., Gmaschitz, T., Greb, P., Karolyi-Ozguer, J. et al. (2019). Highly selective PTK2 proteolysis targeting chimeras to probe focal adhesion kinase scaffolding functions. *J. Med. Chem.* 62, 2508-2520. 10.1021/acs.jmedchem.8b01826 30739444

[JCS261723C103] Poullet, P., Gautreau, A., Kadare, G., Girault, J. A., Louvard, D. and Arpin, M. (2001). Ezrin interacts with focal adhesion kinase and induces its activation independently of cell-matrix adhesion. *J. Biol. Chem.* 276, 37686-37691. 10.1074/jbc.M106175200 11468295

[JCS261723C104] Qiao, M., Zhou, F., Liu, X., Jiang, T., Wang, H., Li, X., Zhao, C., Cheng, L., Chen, X., Ren, S. et al. (2024). Targeting focal adhesion kinase boosts immune response in KRAS/LKB1 co-mutated lung adenocarcinoma via remodeling the tumor microenvironment. *Exp. Hematol. Oncol.* 13, 11. 10.1186/s40164-023-00471-6 38291516 PMC10826079

[JCS261723C105] Qin, Q., Yu, R., Eriksson, J. E., Tsai, H. I. and Zhu, H. (2024). Cancer-associated fibroblasts in pancreatic ductal adenocarcinoma therapy: challenges and opportunities. *Cancer Lett.* 591, 216859. 10.1016/j.canlet.2024.216859 38615928

[JCS261723C106] Roby, K. F., Taylor, C. C., Sweetwood, J. P., Cheng, Y., Pace, J. L., Tawfik, O., Persons, D. L., Smith, P. G. and Terranova, P. F. (2000). Development of a syngeneic mouse model for events related to ovarian cancer. *Carcinogenesis* 21, 585-591. 10.1093/carcin/21.4.585 10753190

[JCS261723C107] Roy-Luzarraga, M. and Hodivala-Dilke, K. (2016). Molecular pathways: endothelial cell FAK-A target for cancer treatment. *Clin. Cancer Res.* 22, 3718-3724. 10.1158/1078-0432.CCR-14-2021 27262114 PMC5386133

[JCS261723C108] Roy-Luzarraga, M., Abdel-Fatah, T., Reynolds, L. E., Clear, A., Taylor, J. G., Gribben, J. G., Chan, S., Jones, L. and Hodivala-Dilke, K. (2020). Association of low tumor endothelial cell pY397-focal adhesion kinase expression with survival in patients with neoadjuvant-treated locally advanced breast cancer. *JAMA Netw. Open* 3, e2019304. 10.1001/jamanetworkopen.2020.19304 33107920 PMC7592032

[JCS261723C109] Roy-Luzarraga, M., Reynolds, L. E., de Luxan-Delgado, B., Maiques, O., Wisniewski, L., Newport, E., Rajeeve, V., Drake, R. J. G., Gomez-Escudero, J., Richards, F. M. et al. (2022). Suppression of endothelial cell FAK expression reduces pancreatic ductal adenocarcinoma metastasis after gemcitabine treatment. *Cancer Res.* 82, 1909-1925. 10.1158/0008-5472.CAN-20-3807 35350066 PMC9381116

[JCS261723C110] Sbrana, F. V., Fiordi, B., Bordini, J., Belloni, D., Barbaglio, F., Russo, L., Scarfo, L., Ghia, P. and Scielzo, C. (2023). PYK2 is overexpressed in chronic lymphocytic leukaemia: A potential new therapeutic target. *J. Cell. Mol. Med.* 27, 576-586. 10.1111/jcmm.17688 36747338 PMC9930416

[JCS261723C111] Schaller, M. D. (2010). Cellular functions of FAK kinases: insight into molecular mechanisms and novel functions. *J. Cell Sci.* 123, 1007-1013. 10.1242/jcs.045112 20332118

[JCS261723C112] Seong, J., Tajik, A., Sun, J., Guan, J. L., Humphries, M. J., Craig, S. E., Shekaran, A., Garcia, A. J., Lu, S., Lin, M. Z. et al. (2013). Distinct biophysical mechanisms of focal adhesion kinase mechanoactivation by different extracellular matrix proteins. *Proc. Natl. Acad. Sci. USA* 110, 19372-19377. 10.1073/pnas.1307405110 24222685 PMC3845171

[JCS261723C113] Serrels, A., Lund, T., Serrels, B., Byron, A., McPherson, R. C., von Kriegsheim, A., Gomez-Cuadrado, L., Canel, M., Muir, M., Ring, J. E. et al. (2015). Nuclear FAK controls chemokine transcription, Tregs, and evasion of anti-tumor immunity. *Cell* 163, 160-173. 10.1016/j.cell.2015.09.001 26406376 PMC4597190

[JCS261723C114] Serrels, B., McGivern, N., Canel, M., Byron, A., Johnson, S. C., McSorley, H. J., Quinn, N., Taggart, D., Von Kreigsheim, A., Anderton, S. M. et al. (2017). IL-33 and ST2 mediate FAK-dependent antitumor immune evasion through transcriptional networks. *Sci. Signal.* 10, eaan8355. 10.1126/scisignal.aan8355 29208683 PMC6128400

[JCS261723C115] Shang, N., Wang, H., Bank, T., Perera, A., Joyce, C., Kuffel, G., Zilliox, M. J., Cotler, S. J., Ding, X., Dhanarajan, A. et al. (2019). Focal adhesion kinase and beta-catenin cooperate to induce hepatocellular carcinoma. *Hepatology* 70, 1631-1645. 10.1002/hep.30707 31069844 PMC6819211

[JCS261723C116] Shapiro, I. M., Kolev, V. N., Vidal, C. M., Kadariya, Y., Ring, J. E., Wright, Q., Weaver, D. T., Menges, C., Padval, M., McClatchey, A. I. et al. (2014). Merlin deficiency predicts FAK inhibitor sensitivity: a synthetic lethal relationship. *Sci. Transl. Med.* 6, 237ra68. 10.1126/scitranslmed.3008639 PMC4165339 24848258

[JCS261723C117] Shen, C. J., Raghavan, S., Xu, Z., Baranski, J. D., Yu, X., Wozniak, M. A., Miller, J. S., Gupta, M., Buckbinder, L. and Chen, C. S. (2011). Decreased cell adhesion promotes angiogenesis in a Pyk2-dependent manner. *Exp. Cell Res.* 317, 1860-1871. 10.1016/j.yexcr.2011.05.006 21640103 PMC3123418

[JCS261723C118] Sieg, D. J., Ilic, D., Jones, K. C., Damsky, C. H., Hunter, T. and Schlaepfer, D. D. (1998). Pyk2 and Src-family protein-tyrosine kinases compensate for the loss of FAK in fibronectin-stimulated signaling events but Pyk2 does not fully function to enhance FAK- cell migration. *EMBO J.* 17, 5933-5947. 10.1093/emboj/17.20.5933 9774338 PMC1170921

[JCS261723C119] Sieg, D. J., Hauck, C. R., Ilic, D., Klingbeil, C. K., Schaefer, E., Damsky, C. H. and Schlaepfer, D. D. (2000). FAK integrates growth-factor and integrin signals to promote cell migration. *Nat. Cell Biol.* 2, 249-256. 10.1038/35010517 10806474

[JCS261723C120] Slack-Davis, J. K., Martin, K. H., Tilghman, R. W., Iwanicki, M., Ung, E. J., Autry, C., Luzzio, M. J., Cooper, B., Kath, J. C., Roberts, W. G. et al. (2007). Cellular characterization of a novel focal adhesion kinase inhibitor. *J. Biol. Chem.* 282, 14845-14852. 10.1074/jbc.M606695200 17395594

[JCS261723C121] Spallarossa, A., Tasso, B., Russo, E., Villa, C. and Brullo, C. (2022). The development of FAK inhibitors: a five-year update. *Int. J. Mol. Sci.* 23, 6381. 10.3390/ijms23126381 35742823 PMC9223874

[JCS261723C122] Stone, R. L., Baggerly, K. A., Armaiz-Pena, G. N., Kang, Y., Sanguino, A. M., Thanapprapasr, D., Dalton, H. J., Bottsford-Miller, J., Zand, B., Akbani, R. et al. (2014). Focal adhesion kinase: an alternative focus for anti-angiogenesis therapy in ovarian cancer. *Cancer Biol. Ther.* 15, 919-929. 10.4161/cbt.28882 24755674 PMC4100993

[JCS261723C123] Sulzmaier, F. J., Jean, C. and Schlaepfer, D. D. (2014). FAK in cancer: mechanistic findings and clinical applications. *Nat. Rev. Cancer* 14, 598-610. 10.1038/nrc3792 25098269 PMC4365862

[JCS261723C124] Takahashi, K., Kanerva, K., Vanharanta, L., Almeida-Souza, L., Lietha, D., Olkkonen, V. M. and Ikonen, E. (2021). ORP2 couples LDL-cholesterol transport to FAK activation by endosomal cholesterol/PI(4,5)P(2) exchange. *EMBO J.* 40, e106871. 10.15252/embj.2020106871 34124795 PMC8281050

[JCS261723C125] Tancioni, I., Uryu, S., Sulzmaier, F. J., Shah, N. R., Lawson, C., Miller, N. L., Jean, C., Chen, X. L., Ward, K. K. and Schlaepfer, D. D. (2014). FAK Inhibition disrupts a beta5 integrin signaling axis controlling anchorage-independent ovarian carcinoma growth. *Mol. Cancer Ther.* 13, 2050-2061. 10.1158/1535-7163.MCT-13-1063 24899686 PMC4126870

[JCS261723C126] Tancioni, I., Miller, N. L., Uryu, S., Lawson, C., Jean, C., Chen, X. L., Kleinschmidt, E. G. and Schlaepfer, D. D. (2015). FAK activity protects nucleostemin in facilitating breast cancer spheroid and tumor growth. *Breast Cancer Res.* 17, 47. 10.1186/s13058-015-0551-x 25880415 PMC4407832

[JCS261723C127] Tang, K. J., Constanzo, J. D., Venkateswaran, N., Melegari, M., Ilcheva, M., Morales, J. C., Skoulidis, F., Heymach, J. V., Boothman, D. A. and Scaglioni, P. P. (2016). Focal adhesion kinase regulates the DNA damage response and its inhibition Radiosensitizes mutant KRAS lung cancer. *Clin. Cancer Res.* 22, 5851-5863. 10.1158/1078-0432.CCR-15-2603 27220963 PMC5122471

[JCS261723C128] Tanjoni, I., Walsh, C., Uryu, S., Tomar, A., Nam, J. O., Mielgo, A., Lim, S. T., Liang, C., Koenig, M., Sun, C. et al. (2010). PND-1186 FAK inhibitor selectively promotes tumor cell apoptosis in three-dimensional environments. *Cancer Biol. Ther.* 9, 764-777. 10.4161/cbt.9.10.11434 20234191 PMC2933317

[JCS261723C129] Tavora, B., Batista, S., Reynolds, L. E., Jadeja, S., Robinson, S., Kostourou, V., Hart, I., Fruttiger, M., Parsons, M. and Hodivala-Dilke, K. M. (2010). Endothelial FAK is required for tumour angiogenesis. *EMBO Mol. Med.* 2, 516-528. 10.1002/emmm.201000106 21154724 PMC3377344

[JCS261723C130] Tavora, B., Reynolds, L. E., Batista, S., Demircioglu, F., Fernandez, I., Lechertier, T., Lees, D. M., Wong, P. P., Alexopoulou, A., Elia, G. et al. (2014). Endothelial-cell FAK targeting sensitizes tumours to DNA-damaging therapy. *Nature* 514, 112-116. 10.1038/nature13541 25079333 PMC4533916

[JCS261723C131] Taylor, K. N. and Schlaepfer, D. D. (2018). Adaptive resistance to chemotherapy, a multi-FAK-torial linkage. *Mol. Cancer Ther.* 17, 719-723. 10.1158/1535-7163.MCT-17-1177 29610281 PMC6538033

[JCS261723C132] Thomas, K. S., Owen, K. A., Conger, K., Llewellyn, R. A., Bouton, A. H. and Casanova, J. E. (2019). Non-redundant functions of FAK and Pyk2 in intestinal epithelial repair. *Sci. Rep.* 9, 4497. 10.1038/s41598-019-41116-1 30872746 PMC6418130

[JCS261723C133] Tomar, A. and Schlaepfer, D. D. (2009). Focal adhesion kinase: switching between GAPs and GEFs in the regulation of cell motility. *Curr. Opin. Cell Biol.* 21, 676-683. 10.1016/j.ceb.2009.05.006 19525103 PMC2754589

[JCS261723C134] Toutant, M., Costa, A., Studler, J. M., Kadare, G., Carnaud, M. and Girault, J. A. (2002). Alternative splicing controls the mechanisms of FAK autophosphorylation. *Mol. Cell. Biol.* 22, 7731-7743. 10.1128/MCB.22.22.7731-7743.2002 12391143 PMC134714

[JCS261723C135] Urciuoli, E. and Peruzzi, B. (2020). Involvement of the FAK network in pathologies related to altered mechanotransduction. *Int. J. Mol. Sci.* 21, 9426. 10.3390/ijms21249426 33322030 PMC7764271

[JCS261723C136] Wang, Y., Douville, C., Chien, Y. W., Wang, B. G., Chen, C. L., Pinto, A., Smith, S. A., Drapkin, R., Chui, M. H., Numan, T. et al. (2024). Aneuploidy landscape in precursors of ovarian cancer. *Clin. Cancer Res.* 30, 600-615. 10.1158/1078-0432.CCR-23-0932 38048050

[JCS261723C137] Wang-Gillam, A., Lim, K. H., McWilliams, R., Suresh, R., Lockhart, A. C., Brown, A., Breden, M., Belle, J. I., Herndon, J., Bogner, S. J. et al. (2022). Defactinib, pembrolizumab, and gemcitabine in patients with advanced treatment refractory pancreatic cancer: A phase I, dose escalation, and expansion study. *Clin. Cancer Res.* 28, 5254-5262. 10.1158/1078-0432.CCR-22-0308 36228156 PMC9772237

[JCS261723C138] Ward, K. K., Tancioni, I., Lawson, C., Miller, N. L., Jean, C., Chen, X. L., Uryu, S., Kim, J., Tarin, D., Stupack, D. G. et al. (2013). Inhibition of focal adhesion kinase (FAK) activity prevents anchorage-independent ovarian carcinoma cell growth and tumor progression. *Clin. Exp. Metastasis* 30, 579-594. 10.1007/s10585-012-9562-5 23275034 PMC3622195

[JCS261723C139] Weis, S. M., Lim, S. T., Lutu-Fuga, K. M., Barnes, L. A., Chen, X. L., Gothert, J. R., Shen, T. L., Guan, J. L., Schlaepfer, D. D. and Cheresh, D. A. (2008). Compensatory role for Pyk2 during angiogenesis in adult mice lacking endothelial cell FAK. *J. Cell Biol.* 181, 43-50. 10.1083/jcb.200710038 18391070 PMC2287283

[JCS261723C140] Worthmuller, J. and Ruegg, C. (2020). The crosstalk between FAK and Wnt signaling pathways in cancer and its therapeutic implication. *Int. J. Mol. Sci.* 21, 9107. 10.3390/ijms21239107 33266025 PMC7730291

[JCS261723C141] Wu, X., Gan, B., Yoo, Y. and Guan, J. L. (2005). FAK-mediated src phosphorylation of endophilin A2 inhibits endocytosis of MT1-MMP and promotes ECM degradation. *Dev. Cell* 9, 185-196. 10.1016/j.devcel.2005.06.006 16054026

[JCS261723C142] Wu, Y., Zhang, K., Seong, J., Fan, J., Chien, S., Wang, Y. and Lu, S. (2016). *In-situ* coupling between kinase activities and protein dynamics within single focal adhesions. *Sci. Rep.* 6, 29377. 10.1038/srep29377 27383747 PMC4935953

[JCS261723C143] Wu, H. J., Hao, M., Yeo, S. K. and Guan, J. L. (2020). FAK signaling in cancer-associated fibroblasts promotes breast cancer cell migration and metastasis by exosomal miRNAs-mediated intercellular communication. *Oncogene* 39, 2539-2549. 10.1038/s41388-020-1162-2 31988451 PMC7310603

[JCS261723C144] Xie, D., Wang, Z., Sun, B., Qu, L., Zeng, M., Feng, L., Guo, M., Wang, G., Hao, J. and Zhou, G. (2023). High frequency of alternative splicing variants of the oncogene Focal Adhesion Kinase in neuroendocrine tumors of the pancreas and breast. *Front. Med.* 15, 907-923. 10.1007/s11684-023-1009-7 37682378

[JCS261723C145] Zaghdoudi, S., Decaup, E., Belhabib, I., Samain, R., Cassant-Sourdy, S., Rochotte, J., Brunel, A., Schlaepfer, D., Cros, J., Neuzillet, C. et al. (2020). FAK activity in cancer-associated fibroblasts is a prognostic marker and a druggable key metastatic player in pancreatic cancer. *EMBO Mol. Med.* 12, e12010. 10.15252/emmm.202012010 33025708 PMC7645544

[JCS261723C146] Zhang, H., Liu, T., Zhang, Z., Payne, S. H., Zhang, B., McDermott, J. E., Zhou, J. Y., Petyuk, V. A., Chen, L., Ray, D. et al. (2016). Integrated proteogenomic characterization of human high-grade serous ovarian cancer. *Cell* 166, 755-765. 10.1016/j.cell.2016.05.069 27372738 PMC4967013

[JCS261723C147] Zhang, B., Zhang, Y., Zhang, J., Liu, P., Jiao, B., Wang, Z. and Ren, R. (2021). Focal adhesion kinase (FAK) inhibition synergizes with KRAS G12C inhibitors in treating cancer through the regulation of the FAK-YAP signaling. *Adv. Sci. (Weinh)* 8, e2100250. 10.1002/advs.202100250 34151545 PMC8373085

[JCS261723C148] Zhang, B., Li, N., Gao, J., Zhao, Y., Jiang, J., Xie, S., Zhang, C., Zhang, Q., Liu, L., Wang, Z. et al. (2024). Targeting of focal adhesion kinase enhances the immunogenic cell death of PEGylated liposome doxorubicin to optimize therapeutic responses of immune checkpoint blockade. *J. Exp. Clin. Cancer Res.* 43, 51. 10.1186/s13046-024-02974-4 38373953 PMC10875809

[JCS261723C149] Zhou, D. W., Fernandez-Yague, M. A., Holland, E. N., Garcia, A. F., Castro, N. S., O'Neill, E. B., Eyckmans, J., Chen, C. S., Fu, J., Schlaepfer, D. D. et al. (2021). Force-FAK signaling coupling at individual focal adhesions coordinates mechanosensing and microtissue repair. *Nat. Commun.* 12, 2359. 10.1038/s41467-021-22602-5 33883558 PMC8060400

[JCS261723C150] Zouq, N. K., Keeble, J. A., Lindsay, J., Valentijn, A. J., Zhang, L., Mills, D., Turner, C. E., Streuli, C. H. and Gilmore, A. P. (2009). FAK engages multiple pathways to maintain survival of fibroblasts and epithelia: differential roles for paxillin and p130Cas. *J. Cell Sci.* 122, 357-367. 10.1242/jcs.030478 19126677 PMC2724727

